# Disturbed flow increases endothelial inflammation and permeability via a Frizzled-4-β-catenin-dependent pathway

**DOI:** 10.1242/jcs.260449

**Published:** 2023-03-24

**Authors:** Matthew Rickman, Mean Ghim, Kuin Pang, Ana Cristina von Huelsen Rocha, Elena M. Drudi, Macià Sureda-Vives, Nicolas Ayoub, Virginia Tajadura-Ortega, Sarah J. George, Peter D. Weinberg, Christina M. Warboys

**Affiliations:** ^1^ Department of Bioengineering, Imperial College London, London SW7 2AZ, UK; ^2^ Translational Health Sciences, Bristol Medical School, Research Floor Level 7, Bristol Royal Infirmary, Bristol BS2 8HW, UK; ^3^ Department of Comparative Biomedical Sciences, Royal Veterinary College, Royal College Street, London NW1 0TU, UK

**Keywords:** Mechanotransduction, Inflammation, Orbital shaker, Atherosclerosis, Adherens junctions, Cytoskeleton

## Abstract

Multidirectional or disturbed flow promotes endothelial dysfunction and is associated with early atherogenesis. Here we investigated the role of Wnt signalling in flow-mediated endothelial dysfunction. The expression of Frizzled-4 was higher in cultured human aortic endothelial cells (ECs) exposed to disturbed flow compared to that seen for undisturbed flow, obtained using an orbital shaker. Increased expression was also detected in regions of the porcine aortic arch exposed to disturbed flow. The increased Frizzled-4 expression in cultured ECs was abrogated following knockdown of R-spondin-3. Disturbed flow also increased the nuclear localisation and activation of β-catenin, an effect that was dependent on Frizzled-4 and R-spondin-3. Inhibition of β-catenin using the small-molecule inhibitor iCRT5 or knockdown of Frizzled-4 or R-spondin-3 resulted in reduced expression of pro-inflammatory genes in ECs exposed to disturbed flow, as did inhibition of WNT5A signalling. Inhibition of the canonical Wnt pathway had no effect. Inhibition of β-catenin also reduced endothelial paracellular permeability; this was associated with altered junctional and focal adhesion organisation and cytoskeletal remodelling. These data suggest the presence of an atypical Frizzled-4-β-catenin pathway that promotes endothelial dysfunction in response to disturbed flow.

## Teaser

**Highlighted Article:** Disturbed flow is a key factor in promoting endothelial dysfunction. Frizzled-4 mediates the pro-inflammatory and barrier-disrupting effects of disturbed flow in endothelial cells via a WNT5A- and β-catenin-dependent mechanism.

## INTRODUCTION

Endothelial cells (ECs) are continuously exposed to haemodynamic wall shear stress (the frictional force per unit area exerted by flowing blood). Surface mechanosensors enable ECs to sense and respond to this shear stress via various mechanosignalling pathways (Givens and Tzima, 2016). The regulation of these pathways differs greatly depending on local vessel geometry and the resulting shear stress to which ECs are exposed, leading in turn to significant differences in EC function. ECs in straight, unbranched regions of arteries experience high-magnitude, pulsatile but uniaxial wall shear stress that is a key determinant of EC homeostasis, activating cytoprotective signalling pathways and promoting the expression of KLF-2, KLF-4, eNOS (or NOS3) and thrombomodulin. Conversely, ECs in atherosclerosis-prone areas within regions of high curvature, branching or bifurcation exhibit endothelial dysfunction, with increased permeability, reduced expression of eNOS and activation of pro-inflammatory signalling pathways (Davies et al., 2013).

The exact type of shear stress that leads to the latter behaviour is controversial. Recent work has challenged the consensus that low time-averaged wall shear stress and a high oscillatory shear index drives EC dysfunction and atherogenesis (Peiffer et al., 2013), and, instead, has highlighted the importance of disturbed and/or multidirectional flow (i.e. high transverse wall shear stress) (Mohamied et al., 2015); however, the precise trigger is still unknown. The mechanisms by which disturbed flow influences EC behaviour are poorly defined, although some signalling pathways have been identified that contribute to aspects of flow-dependent EC dysfunction (Warboys et al., 2014a,b; Mahmoud et al., 2016; Serbanovic-Canic et al., 2017; Alfaidi et al., 2020). Our aim is to better understand mechanosignalling and function in ECs exposed to flow that is multidirectional.

The Wnt signalling pathway is known to regulate responses to mechanical forces in non-vascular mechanoresponsive cells (Warboys, 2018), and a similar role in vascular cells is becoming apparent. Non-canonical Wnt ligands regulate EC polarisation in a flow-dependent manner (Franco et al., 2016), whereas transcriptomic analysis reveals that Wnt pathway genes are significantly enriched in ECs exposed to atherogenic flow environments (Maimari et al., 2016; Bondareva et al., 2019), suggesting a possible role in promoting flow-mediated EC dysfunction (Bondareva et al., 2019). Moreover, low and/or oscillatory shear stress increases β-catenin signalling in ECs, which promotes the activation of NF-κB (Gelfand et al., 2011). Wnt signalling pathways (reviewed in MacDonald et al., 2009) are highly conserved and require the interaction of Wnt with Frizzled receptors on the cell surface. Complexity arises from the presence of multiple Wnt ligands, Frizzled receptors and co-receptors that can associate in various combinations in a context-dependent manner (Dijksterhuis et al., 2014). Canonical Wnt signalling requires the dephosphorylation and stabilisation of β-catenin (CTNNB1), typically through inhibition of the β-catenin destruction complex, allowing β-catenin to translocate to the nucleus and regulate transcription of target genes via interaction with transcription factors such as TCF-4 (MacDonald et al., 2009). In this study, we sought to investigate and dissect the contribution of canonical Wnt signalling pathways in mediating EC dysfunction in response to disturbed flow using an *in vitro* model that is uniquely capable of creating disturbed, multidirectional flow (Ghim et al., 2018; Warboys et al., 2019; Pang et al., 2021).

## RESULTS

### Frizzled-4 expression is increased by disturbed flow and promotes endothelial inflammatory signalling

**Fig. 1.**
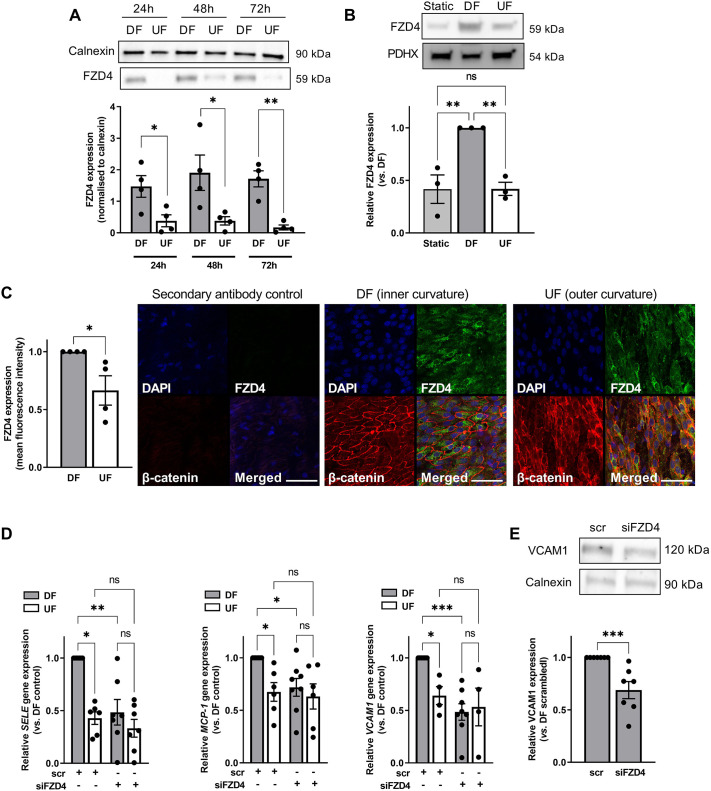
**FZD4 expression is increased in ECs exposed to disturbed flow and regulates pro-inflammatory signalling.** (A,B) Protein lysates were obtained from (A) HAECs exposed to DF and UF for 24–72 h or from (B) HAECs exposed to DF, UF or static conditions for 72 h. FZD4 expression was analysed by western blotting using calnexin (A) or PDHX (B) as a loading control. Data were analysed by two-tailed paired *t*-test at each time point (*n*=4) (A) or one-way ANOVA with Tukey's multiple comparison test (*n*=3) (B). Representative blots are shown in the panels above*.* (C) Aortas from juvenile pigs were fixed and sections cut from regions exposed to DF or UF. Sections were stained with anti-FZD4 and anti-β-catenin antibodies. Nuclei were stained with DAPI. Tissue sections were imaged *en face* and mean fluorescence intensity quantified in three fields of view for each flow region (*n*=4; analysis by Mann–Whitney test; representative images are shown). Scale bars: 50 µm. (D,E) RNA (D) or protein lysates (E) were prepared from ECs transfected with FZD4 siRNA (siFzd4) or scrambled (scr) controls and exposed to DF or UF for 48 h. Gene expression was determined by qRT-PCR using *GAPDH* as a housekeeping gene (*n*=6–8; analysis by Kruskal–Wallis test) (D). Expression of VCAM1 was assessed by western blotting using calnexin as a loading control (*n*=7; analysis by Mann–Whitney test; representative blots are shown in the panel) (E). All data are presented as mean±s.e.m and all *n*-values represent independent biological replicates. ns, not significant; *P<0.05; **P<0.01; ***P<0.001.

Frizzled-4 (*FZD4*) mRNA levels were previously shown to be sensitive to flow conditions in a transcriptomic analysis of the porcine aorta (Serbanovic-Canic et al., 2017). Here, we assessed the expression at the protein level following exposure to different flow conditions using the orbital shaker method. Western blot analysis revealed that disturbed flow (DF) significantly increased the expression of FZD4 relative to undisturbed flow (UF) after 24 h and that this increase was sustained for 72 h of flow exposure (Fig. 1A). Subsequent analysis revealed that DF increases the expression of FZD4 relative to its expression under static conditions and UF, and that its expression levels in ECs exposed to static conditions or UF were not significantly different (Fig. 1B). Demonstrating the potential physiological relevance of this finding, we also observed increased FZD4 receptor expression in the inner curvature of the porcine aortic arch, a region exposed to chronic flow disturbance (Suo et al., 2007; Serbanovic-Canic et al., 2017), compared to its expression in the outer curvature, a region exposed to UF (Fig. 1C).

To determine whether FZD4 mediates endothelial dysfunction under prolonged DF, we transfected ECs with siRNA targeting *FZD4* before exposure to flow. The protein expression of FZD4 was reduced by at least 50% after 48 h of flow compared to its expression in ECs transfected with scrambled RNA (Fig. S2). The expression of Frizzled-5, Frizzled-6 and Frizzled-7 transcripts were unaffected (Fig. S2). Knockdown of *FZD4* resulted in significantly reduced expression of several flow-dependent pro-inflammatory genes (*SELE*, *MCP-1* and *VCAM1*) compared to their expression in scrambled RNA-transfected controls (Fig. 1D). This effect was specific to ECs exposed to DF. As expected, the expression of pro-inflammatory genes was significantly lower in ECs exposed to UF and expression levels were not altered following *FZD4* knockdown. We also observed a reduction in VCAM1 protein in ECs exposed to DF (Fig. 1E). Knockdown of *FZD4* did not alter the expression of the flow-sensitive genes *KLF2* and *NOS3* (Fig. S2), suggesting that ECs remain capable of responding to flow.

### FZD4 expression under disturbed flow is regulated by R-spondin-3

**Fig. 2.**
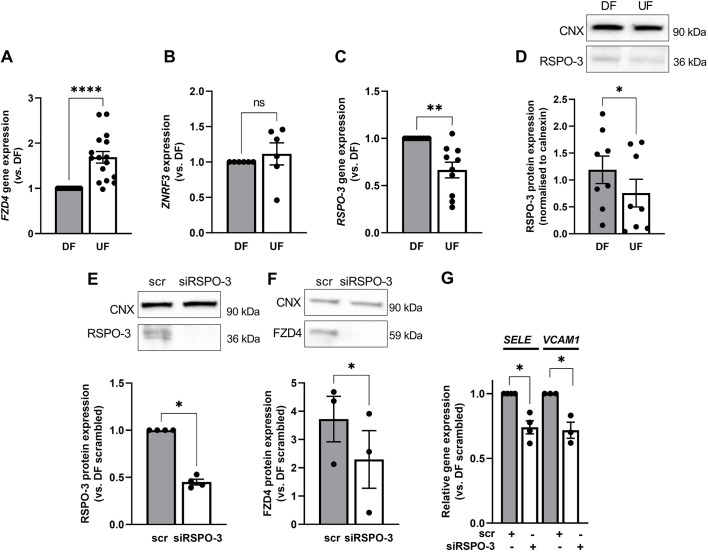
**RSPO-3 expression is increased in ECs exposed to disturbed flow and regulates the expression of FZD4.** (A–D) Lysates were obtained from HAECs exposed to DF and UF for 72 h. (A) RNA lysates were prepared and the expression of (A) *FZD4* (*n*=16), (B) *ZNRF3* (*n*=6) and (C) *RSPO-3* (*n*=10) was determined by qRT-PCR using *GAPDH* as a housekeeping gene (analysis by Wilcoxon matched-pairs signed-rank test). (D) Protein lysates were prepared and analysed by western blotting using an anti-RSPO-3 antibody. Calnexin (CNX) was used as a loading control (*n*=8; analysis by two-tailed paired *t*-test; representative blots are shown in the panel above). (E–G) HAECs were transfected with RSPO-3 siRNA (siRSPO-3) or scrambled controls (scr) and exposed to flow for 48 h. Protein lysates were prepared from ECs exposed to DF and analysed by western blotting using antibodies against RSPO-3 (E) and FZD4 (F). Calnexin was used as a loading control (*n*=3–4; analysis by Mann–Whitney test; representative blots are shown in the panels above). (G) RNA was isolated from ECs exposed to DF and expression of pro-inflammatory genes determined by qRT-PCR using *GAPDH* as a housekeeping gene (*n*=3–4; analysis by Mann–Whitney test). All data are presented as mean±s.e.m. and all *n*-values represent independent biological replicates. ns, not significant; *P<0.05; **P<0.01; ****P<0.0001.

We next sought to determine the mechanism by which DF elevates FZD4 protein expression. Paradoxically, we found that expression of the *FZD4* gene was significantly lower in ECs exposed to DF relative to its expression in ECs exposed to UF (Fig. 2A), pointing towards a post-transcriptional or post-translational mechanism. As FZD4 protein expression can be regulated by lysosomal degradation, mediated by the RNF and ZNRF3 ubiquitin ligases (Koo et al., 2012; Hao et al., 2016), we explored the regulation of this pathway by flow. We found no observable difference between the expression levels of *ZNRF3* under DF and UF (Fig. 2B) but we did observe a significant increase in the expression of R-spondin 3 (RSPO-3), which inhibits ZNRF3 (Hao et al., 2012), in ECs exposed to DF at both the gene (Fig. 2C) and protein (Fig. 2D) levels. The expression of RSPO-1, RSPO-2 and RSPO-4 was undetectable in these cells. These data are consistent with the DF-dependent increase in FZD4 levels being caused by an increased expression of RSPO-3, which would inhibit its degradation. Supporting this view, FZD4 protein expression was significantly reduced in ECs under DF conditions following knockdown of *RSPO-3* (Fig. 2E,F). Moreover, knockdown of *RSPO-3* also reduced the expression of *SELE* and *VCAM1* in ECs exposed to DF (Fig. 2G), mirroring the effects of *FZD4* knockdown.

These data suggest that DF induces pro-inflammatory changes by increasing the expression of FZD4. Our subsequent studies focused on understanding the pathways by which FZD4 determines these and other changes in ECs exposed to DF.

### FZD4 signalling in ECs exposed to disturbed flow is mediated by increased transcriptional activity of β-catenin

FZD4 is known to activate the canonical, β-catenin-dependent Wnt pathway. Previous studies have also determined that β-catenin can be activated in response to low, oscillatory or atherogenic shear stress (Gelfand et al., 2011; Li et al., 2014) but it is not clear whether multidirectional flow (DF) has the same effects. We therefore examined whether the anti-inflammatory effects of *FZD4* knockdown under DF conditions were associated with altered β-catenin signalling. Analysis of fractionated lysates revealed that total β-catenin expression was significantly increased in both cytosolic and nuclear fractions of ECs exposed to DF compared to its expression in ECs exposed to UF, similar to results reported for low and/or oscillatory shear stress (Gelfand et al., 2011; Li et al., 2014). Nuclear expression of the active, dephosphorylated form of β-catenin was also significantly increased in ECs exposed to DF (Fig. S3). Using a reporter assay, we demonstrated that DF (for 1–48 h) significantly increased reporter activity relative to UF, indicating increased β-catenin-mediated transcriptional activity. Reporter activity in ECs exposed to DF also increased over time (Fig. S3). These data support the findings of previous studies that atherogenic flow conditions activate β-catenin signalling (Gelfand et al., 2011; Li et al., 2014).

**Fig. 3.**
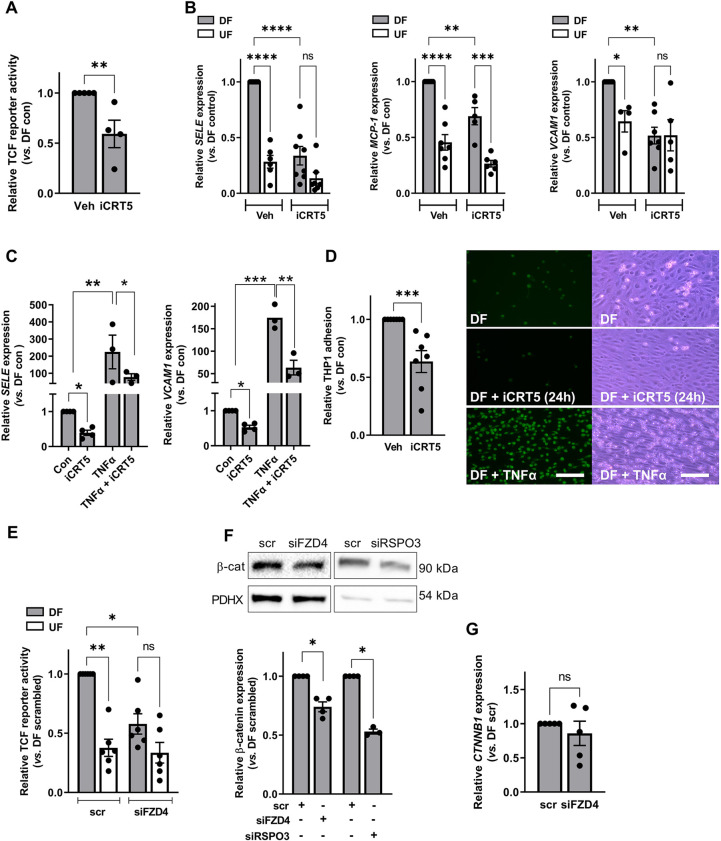
**β-catenin activity is increased in ECs exposed to disturbed flow in a FZD4-dependent manner.** (A–D) HAECs were exposed to flow for 72 h and treated with iCRT5 (50 µM) for the last 24 h of flow exposure. ‘Veh’, vehicle control. (A) HAECs were transfected with TCF reporter constructs 24 h prior to flow exposure. Lysates were prepared from cells exposed to DF and firefly and *Renilla* luciferase activity was recorded. Ratios were corrected for the protein content of lysates. Results shown are relative to the DF control (*n*=4; analysis by Mann–Whitney test). (B,C) RNA was harvested from ECs exposed to DF and UF and gene expression determined by qRT-PCR using *GAPDH* as a housekeeping gene (*n*=4–8; analysis by two-way ANOVA with Tukey's multiple comparison test). (C) HAECs were treated with TNFα for the final 24 h of flow exposure. RNA was harvested from ECs exposed to DF (*n*=3–4; analysis by one-way ANOVA with Tukey's multiple comparison test). Con, control. (D) The number of adherent calcein-labelled THP-1 monocytes was determined in four fields of view following treatment with iCRT5 for the final 24 h of flow exposure or for the full duration. Representative fluorescence images for DF control, iCRT5 (24 h) and TNFα (positive control) are shown; phase-contrast images are also included to show the presence of the intact EC monolayer. Scale bar: 200 µm. Results are shown relative to untreated controls (*n*=7; analysis by Mann–Whitney test). (E) HAECs were transfected with FZD4 siRNA (siFzd4) or scrambled control plus TCF reporter constructs and exposed to flow for 48 h. Lysates were prepared as for A. Results are shown relative to DF control (*n*=6; analysis by Kruskal–Wallis test). (F,G) HAECs were transfected with FZD4 or RSPO-3 siRNA or scrambled control and exposed to flow for 48 h. (F) Lysates were prepared from ECs exposed to DF and analysed by western blotting using an anti-β-catenin antibody. PDHX was used as a loading control (*n*=3–4; analysis by Mann–Whitney test). (G) Transcript levels of β-catenin (*CTNNB1*) were assessed by qRT-PCR using *GAPDH* as a housekeeping gene (*n*=5; analysis by Mann–Whitney test). All data are presented as mean±s.e.m. and all *n*-values represent independent biological replicates. ns, not significant; *P<0.05; **P<0.01; ***P<0.001; ****P<0.0001.

Previous studies have also shown that β-catenin can activate inflammatory pathways under atherogenic shear stress (Gelfand et al., 2011). We sought to confirm these findings in ECs exposed to DF using iCRT5, a small-molecule inhibitor of β-catenin transcriptional activity (Gonsalves et al., 2011). Addition of iCRT5 to ECs for the last 24 h of flow exposure significantly inhibited β-catenin transcriptional activity (Fig. 3A) and reduced the expression of *SELE*, *MCP-1* and *VCAM1* transcripts specifically under DF conditions relative to their expression in vehicle-treated controls (Fig. 3B). The addition of iCRT5 had no effect on pro-inflammatory gene expression in ECs exposed to UF (Fig. 3B). Similar results were obtained when iCRT5 was added for the duration of flow or when β-catenin expression was reduced following transfection of ECs with siRNA targeting β-catenin (Fig. S4). Treatment with iCRT5 for the final 24 h of flow exposure also significantly attenuated the upregulation of pro-inflammatory genes induced by TNFα (TNF) (Fig. 3C). Furthermore, addition of iCRT5 for the final 24 h of flow exposure resulted in significantly reduced adhesion of monocytes to ECs exposed to DF (Fig. 3D). Similar results were obtained when iCRT5 was added for the duration of flow (data not shown). These data support the idea that β-catenin modulates inflammatory activation in response to atheroprone flow and they mirror the effects of *FZD4* or *RSPO-3* knockdown.

Importantly, reduction of *FZD4* expression by siRNA interference resulted in a significant decrease in β-catenin transcriptional activity under DF conditions. No effect was observed in ECs exposed to UF (Fig. 3E). *FZD4* knockdown was also associated with reduced β-catenin protein levels in whole-cell lysates (Fig. 3F). Transcript levels of β-catenin were unaffected (Fig. 3G). Moreover, knockdown of *RSPO-3* also reduced the expression of β-catenin in whole-cell lysates under DF conditions (Fig. 3F). These data suggest that FZD4-dependent activation of β-catenin mediates, at least in part, the pro-inflammatory effects of DF.

### Inhibition of WNT5A signalling reduces disturbed flow-dependent activation of β-catenin and attenuates inflammatory signalling

**Fig. 4.**
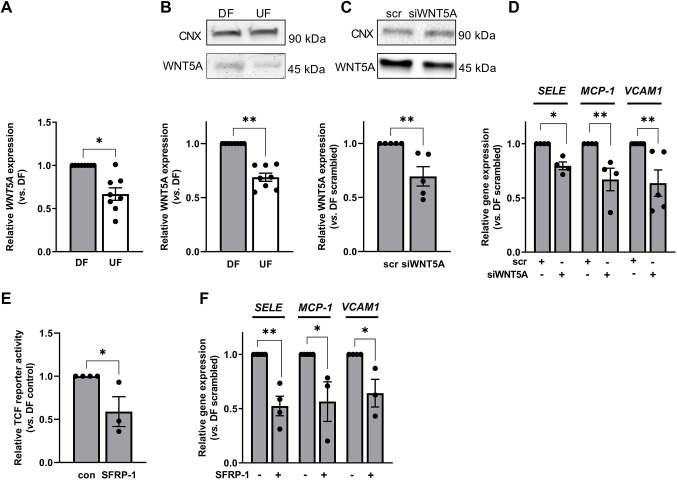
**WNT5A expression is increased in ECs exposed to disturbed flow.** (A,B) HAECs were exposed to flow for 72 h and the expression of WNT5A was quantified in cells exposed to DF or UF by (A) qRT-PCR using *GAPDH* as a housekeeping gene (*n*=8; analysis by Wilcoxon signed-rank test) or (B) western blot using an anti-WNT5A antibody. Calnexin (CNX) was used as a loading control (*n*=8; analysis by Wilcoxon signed-rank test). (C,D) HAECs were transfected with WNT5A siRNA (siWnt5a) or scrambled controls and exposed to flow for 48 h. (C) WNT5A expression was assessed by western blotting using calnexin as a loading control (*n*=5; analysis by Mann–Whitney test). (D) Gene expression was determined by qRT-PCR using *GAPDH* as a housekeeping gene (*n*=4–5; analysis by Mann–Whitney test). (E,F) HAECs were exposed to flow for 72 h in the presence of sFRP-1 (200 µg ml^−1^) for the duration of flow exposure. (E) HAECs were transfected with TCF reporter constructs 24 h prior to flow exposure. Lysates were prepared from cells exposed to DF and firefly and *Renilla* luciferase activity was recorded. Ratios were corrected for protein content of lysates. Results are shown relative to the DF control (*n*=3; analysis by Mann–Whitney test). (F) Gene expression was determined by qRT-PCR using *GAPDH* as a housekeeping gene (*n*=3–4; analysis by Mann–Whitney test). All data are presented as mean±s.e.m. and all *n*-values represent independent biological replicates. *P<0.05; **P<0.01.

As Frizzled receptors are commonly activated by Wnt ligands, we assessed the role of WNT5A in mediating the response to DF. We focused on WNT5A because it is known to promote inflammatory signalling in ECs (Bretón-Romero et al., 2016). We found significantly increased expression of WNT5A in ECs exposed to DF compared to UF at both the transcript (Fig. 4A) and protein (Fig. 4B) levels. Transfection with *WNT5A* siRNA resulted in a 35–40% reduction in WNT5A expression (Fig. 4C) and reduced the expression of *SELE*, *MCP-1* and *VCAM1* (Fig. 4D).

These data suggest that WNT5A can promote endothelial dysfunction under DF conditions. This interpretation was supported by experiments in which ECs were treated with SFRP-1, which blocks the interaction of WNT5A with Frizzled receptors (Dufourcq et al., 2008) and is shown here to reduce β-catenin reporter activity (Fig. 4E). Similar to knockdown of *WNT5A* and *FZD4* and inhibition of β-catenin signalling, SFRP-1 treatment for 24 h significantly reduced the expression of *SELE* and *MCP-1* in ECs exposed to DF (Fig. S6). Interestingly, the inhibition was greater when cells were exposed to SFRP-1 for 72 h and the expression of *VCAM1* was also significantly reduced (Fig. 4F).

### FZD4 signalling in ECs exposed to disturbed flow acts via GSK3β but is independent of LRP6

**Fig. 5.**
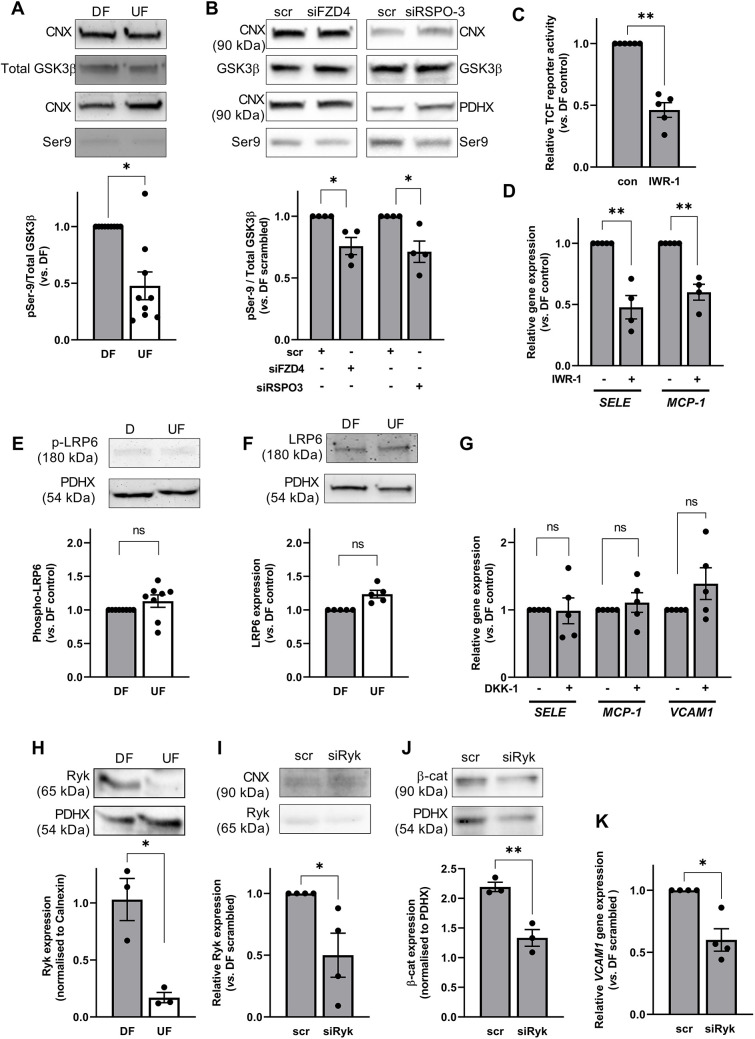
**Disturbed flow inhibits the β-catenin destruction complex in a FZD4-, RSPO-3- and Ryk-dependent manner.** (A,B) GSK3β phosphorylation was quantified by western blotting using anti-phospho GSK3β Ser9 and anti-GSK3β (total) antibodies in protein lysates from HAECs exposed to (A) DF and UF for 72 h or (B) DF for 48 h following transfection with FZD4 or RSPO-3 siRNA or scrambled controls. Calnexin (CNX) was used as a loading control. Results are shown relative to the DF control (A, *n*=9, analysis by Wilcoxon signed-rank test; and B, *n*=4, analysis by Mann–Whitney test). (C,D) HAECs were exposed to flow for 72 h and IWR-1 (10 µM) was added for the final 24 h of flow exposure. (C) HAECs were transfected with TCF reporter constructs 24 h prior to flow exposure. Lysates were prepared from cells exposed to DF and firefly and *Renilla* luciferase activity was recorded. Ratios were corrected for protein content of lysates. Results are shown relative to DF control (*n*=5; analysis by two-tailed unpaired *t*-test). (D) Gene expression was determined by qRT-PCR using *GAPDH* as a housekeeping gene (*n*=4; analysis by two-tailed unpaired *t*-test). (E,F) Lysates were obtained from ECs exposed to DF and UF for 72 h and (E) phosphorylated (*n*=8) or (F) total (*n*=5) LRP6 levels assessed by western blot using PDHX as a loading control (analysis by Wilcoxon signed-rank test). (G) HAECs were exposed to flow for 72 h and treated with DKK-1 (250 ng ml^−1^) f for the last 24 h of flow exposure. RNA was harvested from ECs exposed to DF and gene expression assessed by qRT-PCR using *GAPDH* as a housekeeping gene (*n*=4–5; analysis by Mann–Whitney test). (H–K) Lysates were obtained (H) from HAECs exposed to DF and UF for 72 h or (I–K) from HAECs exposed to DF for 48 h following transfection with Ryk siRNA (siRyk) or scrambled controls. (H–J) Expression of proteins was determined by western blotting using antibodies targeting (H,I) Ryk (*n*=3–4) or (J) β-catenin (*n*=3). PDHX or calnexin was used as a loading control (representative blots are shown in the panels above; *n*=3-4; analysis by Mann–Whitney test). (K) *VCAM1* expression was assessed by qRT-PCR using *GAPDH* as a housekeeping gene (*n*=4; analysis by Mann–Whitney test). All data are presented as mean±s.e.m. and all *n*-values represent independent biological replicates. ns, not significant; *P<0.05; **P<0.01.

The data presented so far suggests that EC dysfunction under DF conditions can be promoted via WNT5A-FZD4 signalling. The requirement for β-catenin is consistent with the involvement of a canonical (β-catenin-dependent) Wnt signalling pathway, and this is supported by the finding that phosphorylation of GSK3β (encoded by *GSK3B*) Ser9 was increased in ECs by exposure to DF compared to its levels following UF (Fig. 5A). Phosphorylation of the Ser9 residue inhibits GSK3β and is a necessary step in the activation of β-catenin; it results in inhibition of the β-catenin destruction complex, leading to the nuclear localisation of dephosphorylated (active) β-catenin. GSK3β phosphorylation on Ser9 was significantly reduced following knockdown of *FZD4* or *RSPO-3* (Fig. 5B), suggesting a role for the destruction complex in mediating their effects on β-catenin signalling in ECs exposed to DF. We consequently investigated whether stabilisation of the β-catenin destruction complex affected the activation of β-catenin and the phenotype of ECs exposed to DF. We found that treating the cells with the Wnt pathway inhibitor IWR-1 (which stabilises Axin2 levels) inhibited the transcriptional activity of β-catenin and reduced the expression of *SELE* and *MCP-1* under DF conditions, mimicking the effects of *WNT5A* and *FZD4* knockdown and inhibition of β-catenin signalling (Fig. 5C,D). These data are consistent with a role for activation of a FZD4-dependent canonical Wnt pathway in cells exposed to DF.

To test this further, we assessed the activation of LRP6 (a hallmark of the canonical Wnt pathway) under flow conditions. Surprisingly, we found no evidence of higher LRP6 phosphorylation under DF than under UF (Fig. 5E). Total levels of LRP6 protein were also unchanged (Fig. 5F). Moreover, inhibiting canonical Wnt signalling by treating ECs with DKK-1 (which blocks the interaction of LRP and Frizzled receptors) for 24 h did not have the expected effect on the expression of pro-inflammatory genes (Fig. 5G) or THP-1 monocyte adhesion (data not shown). A similar lack of effects was observed when DKK-1 was added for the duration of flow exposure (data not shown).

Because these data suggest that FZD4 activates β-catenin independently of LRP signalling, we investigated alternative Wnt pathway components that could mediate the effects of FZD4 activation and found that Ryk expression was significantly increased in ECs exposed to DF for 72 h (Fig. 5H). Transfection with *RYK* siRNA resulted in a significant reduction in Ryk expression (Fig. 5I) and was associated with reduced levels of β-catenin in ECs exposed to DF (Fig. 5J), consistent with a role in regulating β-catenin under DF conditions. Moreover, knockdown of *RYK* reduced the expression of *VCAM1* in cells exposed to DF (Fig. 5K).

### Inhibiting β-catenin activity reduces the elevated paracellular permeability seen in monolayers exposed to disturbed flow

**Fig. 6.**
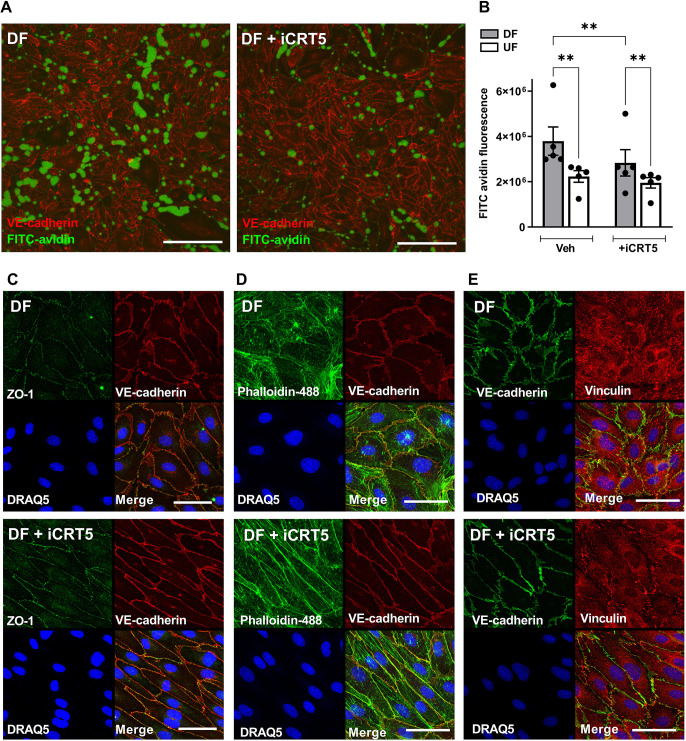
**β-catenin activity increases permeability in ECs exposed to disturbed flow and alters cytoskeletal and junctional organisation.** (A–E) HAECs were exposed to flow for 72 h and treated with iCRT5 (50 µM) for the last 24 h of flow exposure. (A) FITC–avidin was added to monolayers immediately after flow cessation. Images show areas where FITC–avidin binds to biotinylated gelatin underlying ECs. Cells were counterstained with an anti-VE-cadherin antibody (images shown are maximum projections of *z*-stacks). Scale bars: 200 µm. (B) Accumulation of FITC–avidin was quantified by determining the intensity of FITC–avidin in maximum projections and shown relative to the DF vehicle control (*n*=5; analysis by two-way ANOVA with Tukey's multiple comparison test). (C–E) ECs were fixed and stained with an anti-VE-cadherin antibody and DRAQ5 nuclear stain with (C) an anti-ZO-1 antibody, (D) Alexa Fluor 488–Phalloidin or (E) an anti-vinculin antibody (representative images from four independent experiments). Scale bars: 50 µm. All data are presented as mean±s.e.m. and all *n*-values represent independent biological replicates. **P<0.01.

Aside from increased pro-inflammatory signalling, DF-induced endothelial dysfunction is also associated with increased endothelial permeability (Wu et al., 2015; Ghim et al., 2017; Lyu et al., 2019; Alfaidi et al., 2020; Yang et al., 2020). DF increases monolayer permeability to FITC–avidin compared to that following UF or static culture by a paracellular route (Ghim et al., 2017, 2022). As inhibition of β-catenin signalling altered DF-induced inflammatory activation, we assessed whether inhibition could also alter barrier function in ECs exposed to DF. Inhibition of β-catenin transcriptional activity using iCRT5 significantly reduced permeability to FITC–avidin under DF (Fig. 6A,B). Permeability of ECs exposed to UF was not affected (Fig. 6B). Closer inspection showed that transport at bicellular junctions appeared to be particularly reduced (Fig. S5). Immunostaining of ZO-1 and VE-cadherin (CDH5) revealed a clear change in junctional organisation following inhibition of β-catenin in ECs exposed to DF, along with increased expression of ZO-1 (Fig. 6C; quantified in Fig. S5). Junctions appeared disorganised and irregular in control ECs exposed to DF but were more organised following treatment with iCRT5 (Fig. 6C). The morphology of ECs exposed to DF was also altered by iCRT5: the cells appeared more consistently oriented and more elongated (representative images in Fig. 6C–E). Quantification of the length-to-width ratio confirmed the latter observation (Fig. S5; UF included for reference).

**Fig. 7.**
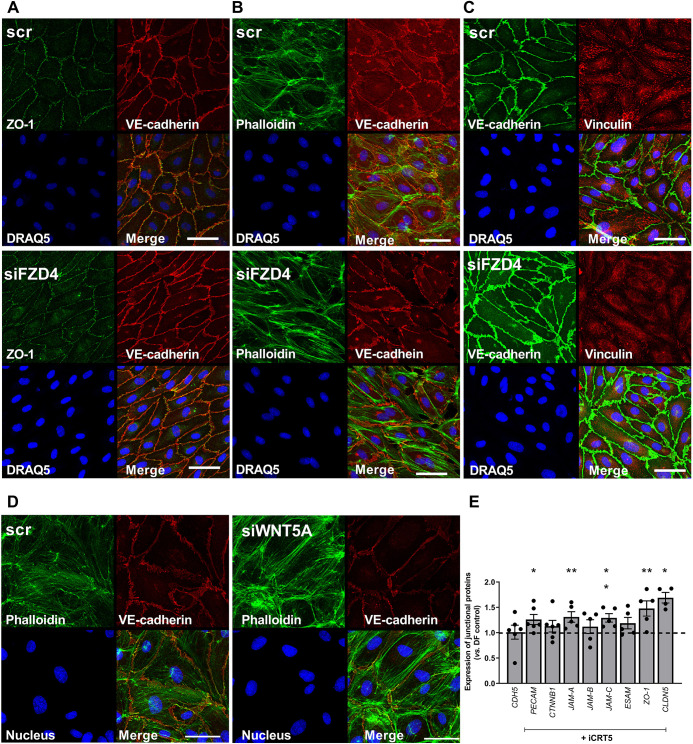
**Knockdown of *FZD4* or *WNT5A* alters organisation of the cytoskeleton and vinculin in ECs exposed to disturbed flow.** (A–D) HAECs were exposed to disturbed flow for 48 h following transfection with siRNAs targeting (A–C) FZD4 (siFzd4) or (D) WNT5A (siWnt5a) and compared to scrambled RNA-transfected control (scr). ECs were fixed and stained with an anti-VE-cadherin antibody and DRAQ5 nuclear stain with (A) anti-ZO-1, (B,D) Alexa Fluor 488–Phalloidin or (C) anti-vinculin antibody (representative images from four independent experiments). Scale bars: 50 µm. (E) HAECs were exposed to flow for 72 h and treated with iCRT5 (50 µM) for the last 24 h of flow exposure. RNA was harvested from ECs exposed to DF and gene expression assessed by qRT-PCR using *GAPDH* as a housekeeping gene (*n*=4–6; analysis by Mann–Whitney test). All data are presented as mean±s.e.m. and all *n*-values represent independent biological replicates. *P<0.05; **P<0.01.

We also assessed the effects of β-catenin inhibition on the actin cytoskeleton in cells exposed to DF. Staining with phalloidin revealed striking differences after treatment with iCRT5. There was a reduction in the number of stress fibres and an increase in junctional (cortical) staining that is typically associated with stabilisation of junctions and reduced permeability (Fig. 6D) (McCue et al., 2004). The distribution of vinculin under DF was also modified by iCRT5: there was greater localisation around junctions compared to the apparent localisation to focal adhesions in untreated cells (Fig. 6E). There was a similar change in morphology, junctional organisation, cytoskeletal architecture and vinculin localisation following knockdown of *FZD4* (Fig. 7A–C).

Knockdown of *WNT5A* also resulted in reduced numbers of stress fibres, increased cortical actin and apparent elongation of ECs exposed to DF, similar to the effects observed with knockdown of *FZD4* or inhibition of β-catenin signalling with iCRT5 (Fig. 7D). Furthermore, SFRP-1 and IWR-1 treatment also resulted in morphological, junctional and cytoskeletal changes similar to those observed previously (Figs S6 and S7). DKK-1 had no effect on the length-to-width ratio or cytoskeletal or junctional organisation (Figs S5 and S7).

Increased permeability can be associated with ECs undergoing endothelial–mesenchymal transition and this process is known to be increased by DF (Mahmoud et al., 2017) and by canonical Wnt signalling (Liebner et al., 2004). No evidence was found here to suggest that this was a contributing factor as iCRT5 treatment or *FZD4* knockdown had no effect on the expression of genes associated with the endothelial–mesenchymal transition (see Fig. S5). We subsequently investigated whether inhibiting β-catenin signalling affected the expression of junctional molecules. iCRT5 did not induce changes in the transcript levels of VE-cadherin (*CDH5*), *ESAM*, *JAM-B* (also known as *JAM2*) or *CTNNB1* in ECs exposed to DF. However, it did increase expression of *PECAM-1*, *JAM-A* (*F11R*), *JAM-C* (*JAM3*), *ZO-1* (*TJP1*) and *CLDN5* (Fig. 7E). Taken together, these data suggest that inhibiting β-catenin signalling increases the expression of junctional proteins and stabilises cell–cell junctions in ECs exposed to DF and that this might account for the enhanced barrier properties observed in the presence of iCRT5.

## DISCUSSION

**Fig. 8.**
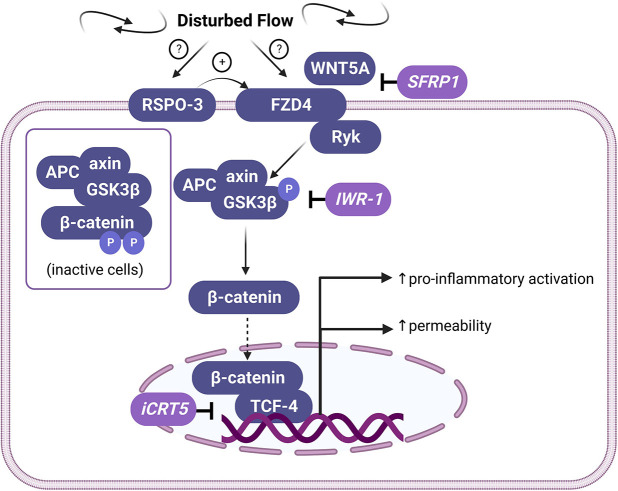
**Proposed pathway of disturbed flow-dependent FZD4-β-catenin signalling.** Disturbed flow increases the expression of FZD4 via an RSPO-3-dependent mechanism. Activation of FZD4 by WNT5A inhibits the GSK3β-axin-APC destruction complex, promoting the stabilisation and nuclear translocation of β-catenin. Increased transcriptional activity of β-catenin promotes endothelial dysfunction via increased pro-inflammatory signalling and barrier disruption. The targets of inhibitors used in this study are shown at relevant points in the pathway. Image created with BioRender.com and published with a BioRender content licence for use in academic journals.

Here, we provide evidence of a novel FZD4-dependent signalling pathway, summarised in Fig. 8, that is activated in ECs in response to DF. The expression of FZD4 is significantly higher under DF than under UF, and this is dependent on increased expression of RSPO-3. In response to DF, FZD4 increases the transcriptional activation of β-catenin independently of LRP signalling. β-catenin promotes the inflammatory activation and barrier disruption associated with DF.

The expression of FZD4 in the developed arterial circulation has been demonstrated previously (Descamps et al., 2012), but to our knowledge, this is the first evidence that FZD4 protein expression is elevated in response to DF. *FZD4* transcript levels decreased under the same conditions. This observation is in line with data obtained following surgical induction of flow disturbance in mouse carotid arteries (Ni et al., 2010), but a second transcriptomic analysis found increased *FZD4* transcript levels in the inner curvature of the porcine aortic arch; this might reflect an adaptation to chronic DF exposure (Serbanovic-Canic et al., 2017). Regardless of the apparent discrepancies in transcript levels between earlier studies, we found increased FZD4 protein in the inner curvature of the porcine aortic arch, which is chronically exposed to DF, suggesting that the increased protein expression in our cell culture model is physiologically relevant.

We show here that FZD4 contributes to the pro-inflammatory activation of ECs under DF. Interestingly, FZD4 is also required for retinal vascular development and regulates arterial organisation in response to hindlimb ischaemia (Descamps et al., 2012). In these experiments, FZD4 was shown to increase proliferation and migration, although these studies were carried out under static conditions (Descamps et al., 2012). FZD4 function therefore appears to be context and stimulus dependent – although it can play a homeostatic role, it also appears to promote endothelial dysfunction in ECs exposed to DF.

As FZD4 protein levels were elevated under DF without any concomitant rise in transcript levels, we investigated other mechanisms of regulation. The E3 ubiquitin ligases ZNRF3 and RNF43 target Frizzled for lysosomal degradation and thus negatively regulate Frizzled signalling (Hao et al., 2012). We did not observe any change in expression of these enzymes under flow conditions. However, we did find a significant increase in the expression of RSPO-3, which inhibits the activity of ZNRF3 (Hao et al., 2012), in ECs exposed to DF. This plausibly explains the increase in FZD4. Knockdown of *RSPO-3* significantly lowered the expression of FZD4 in cells exposed to DF and reduced the inflammatory activation induced by this type of flow, further supporting a role for RSPO-3 in the regulation of FZD4 under DF. The mechanism by which RSPO-3 is increased by DF is not currently known and will be an important area for future study.

Although we have shown that reducing FZD4 attenuates DF-induced inflammatory activation, the mechanisms by which FZD4 signalling is activated are not fully defined. One possibility is that, as a G protein-coupled receptor (GPCR), FZD4 might be directly activated by mechanical force, as has been demonstrated for other GCPRs (Hu et al., 2021). FZD4 might also require ligand binding as has been demonstrated in bone mesenchymal stem cells, in which WNT5A is required for the activation of FZD4 in response to mechanical forces (Gu et al., 2018). Interestingly, deletion of WNT5A increases the sensitivity of ECs to laminar shear stress, lowering the threshold at which ECs polarise against flow direction (Franco et al., 2016), although the role of WNT5A in regulating responses to non-uniform shear stress have not previously been explored.

Our data support a role for WNT5A in mediating DF-induced inflammatory activation: the expression of WNT5A was increased in ECs exposed to DF and knockdown of *WNT5A* reduced the expression of pro-inflammatory genes under DF. The mechanism by which WNT5A expression is increased by DF was not determined and is an area of future study. Further evidence for the importance of ligand-mediated activation of FZD4 is provided by data showing that DF-induced activation of β-catenin and pro-inflammatory signalling is also reduced following treatment with SFRP-1, which binds and antagonises FZD4 (Dufourcq et al., 2008), although it should be noted that SFRP-1 might have functions at other Frizzled receptors (Dufourcq et al., 2008; Pereira et al., 2008)*.* Our data support the finding that WNT5A promotes canonical Wnt signalling when FZD4 expression is high (Mikels and Nusse, 2006).

WNT5A has previously been shown to promote endothelial dysfunction in static ECs (Bretón-Romero et al., 2016; Cho et al., 2018) via a JNK-dependent pathway (Bretón-Romero et al., 2016). We found no evidence of JNK activation in cells exposed to DF (see Fig. S8), suggesting that non-canonical WNT5A-JNK signalling is not important in mediating responses to DF. Our data instead support a role for a canonical Wnt pathway as knockdown of *FZD4* and *RSPO-3* reduced the activation of β-catenin in ECs exposed to DF. Our observation of increased GSK3β under DF also indicates the presence of a flow-sensitive canonical pathway. However, we could not detect the phosphorylation of LRP6 that is typically associated with canonical Wnt signalling. Furthermore, inhibition of the canonical pathway using DKK-1 (which blocks the interaction between Frizzled and LRP co-receptors) had no effect on EC responses to DF. It is possible that upon stimulation, FZD4 directly activates the β-catenin destruction complex, independently of LRP6. This could occur as a result of dimerisation of FZD4 receptors and consequent clustering of Dishevelled (DVL) proteins (Gammons et al., 2016), leading to the dimerisation and polymerisation that is necessary for activation of the axin degradasome (Gammons and Bienz, 2018). Alternatively, WNT5A-FZD4 signalling might occur independently of DVL, as has been demonstrated during neurite outgrowth (Bian et al., 2015).

Intriguingly, it has also been documented that WNT5A can promote endothelial dysfunction via the Wnt co-receptor Ryk, an atypical receptor kinase that lacks kinase activity (Skaria et al., 2017). Ryk can associate with Frizzled receptors and DVL in 293T cells and this is required for transcriptional activity of TCF-4 (Lu et al., 2004). However, a direct interaction with Wnt pathway components has not previously been demonstrated in ECs. We demonstrated here that Ryk expression is increased in ECs exposed to DF and that depletion of Ryk reduces GSK3β phosphorylation and VCAM1 expression under DF. It is therefore possible that Ryk is recruited to FZD4 receptors upon ligation by WNT5A and contributes to the activation of β-catenin. Taken together, our data suggest the presence of an atypical Wnt signalling pathway that is activated by DF and promotes endothelial dysfunction. Interestingly, Gelfand et al. (2011) also provide evidence that PECAM-1 plays a role in the regulation of β-catenin under atherogenic flow conditions and this could be due to phosphorylation and consequent inhibition of GSK3β (Biswas et al., 2006); DF-induced activation of β-catenin is thus complex and might require multiple converging pathways*.*

We have shown here that the increased expression of RSPO-3 and FZD4 in ECs exposed to DF results in increased nuclear translocation and transcriptional activation of β-catenin. Similar findings were previously obtained in ECs exposed to low and oscillatory shear stress for 6–24 h (Gelfand et al., 2011; Li et al., 2014), suggesting that activation of β-catenin is common under atherogenic flow conditions. We also demonstrated that increased transcriptional activity of β-catenin resulted in increased expression of pro-inflammatory genes (*VCAM1*, *MCP-1* and *SELE*) and increased adhesion of THP-1 monocytes in ECs exposed to DF. Interestingly, RSPO-3 has also been shown to enhance non-canonical Wnt-Ca^2+^-NFAT signalling in ECs (Scholz et al., 2016). The regulation of non-canonical pathways by FZD4 under flow conditions is an important area for future research.

In addition to its actions on pro-inflammatory signalling, our data reveal important effects of flow-activated Wnt signalling and β-catenin transcriptional activity on cell morphology, cytoskeletal organisation and permeability. Ghim et al. (2017, 2022) recently demonstrated that monolayers exposed to DF exhibit increased permeability to FITC–avidin, which we confirmed here. We additionally found that inhibiting β-catenin transcriptional activity with a small-molecule inhibitor significantly reduced endothelial permeability. It is likely that these effects are specific to arterial ECs as there appear to be key differences in Wnt-β-catenin signalling between the systemic circulation and the blood–brain or blood–retinal barrier. β-catenin plays a critical role in the development of the blood–brain barrier but appears to have little effect on embryonic vascular development in other arterial beds (Cattelino et al., 2003; Daneman et al., 2009). Moreover, conditional endothelial deletion of β-catenin impairs the integrity of the blood–brain barrier, whereas it has no effect on the integrity of the pulmonary vasculature (Tran et al., 2016).

ECs exposed to DF exhibited a randomly oriented cobblestone morphology and a disorganised actin cytoskeleton with numerous stress fibres. Immunostaining for ZO-1, PECAM-1 and VE-cadherin revealed irregular and disorganised junctions, in line with other observations (Alfaidi et al., 2020). Inhibition of the flow-dependent Wnt pathway at various levels appeared to promote the formation of more organised junctions, plausibly accounting for the reduction in permeability it caused. We also observed a significant increase in *JAM-A*, *JAM-C*, *CLDN5* and *ZO-1* transcript levels following inhibition of β-catenin transcriptional activity. A role for ZO-1 in maintaining VE-cadherin function has been seen in ECs cultured under static conditions: ZO-1 increases tensile force on VE-cadherin, promotes the formation of cortical actomyosin structures and recruits vinculin to adherens junctions (Tornavaca et al., 2015). We also demonstrated in this study that blockade of the DF-induced Wnt pathway is associated with redistribution of vinculin to cell–cell contacts. This is consistent with other evidence suggesting that vinculin binds to VE-cadherin and stabilises adherens junctions during force-dependent remodelling (Huveneers et al., 2012).

Blockade of the Wnt pathway and β-catenin transcriptional activity was also associated with EC elongation and alignment, accompanied by the formation of the dense cortical actin ring typically observed in ECs exposed to unidirectional shear stress (Birukov et al., 2002; McCue et al., 2004) and associated with enhanced barrier function (Garcia et al., 2001). Recent studies provide evidence that ECs re-orient and align themselves to minimise transverse wall shear stress (Arshad et al., 2021) and that ECs fail to remodel when transverse wall shear stress is high, resulting in inflammatory activation (Wang et al., 2012) and increased permeability (Ghim et al., 2017). It is known that unidirectional shear stress induces cytoskeletal remodelling and consequent permeability reduction via rapid activation and translocation of Rac and cortactin to cell–cell junctions (Birukov et al., 2002), along with paxillin and FAK (Shikata et al., 2003). However, the mechanisms governing permeability and cytoskeletal organisation under DF are less well defined, although there appears to be a requirement for the activation of p21–activated kinase (PAK) (Alfaidi et al., 2020). Here, we report that following inhibition of the flow-dependent Wnt pathway at various levels, ECs appeared to undergo dramatic remodelling of junctions and the cytoskeleton, even though transverse wall shear stress remained high. Several Wnt pathway components can interact with and regulate the cytoskeleton (Lai et al., 2009), but our data point towards transcriptional regulation of cytoskeletal components, e.g. ZO-1 or other unidentified effectors. It is also possible that blocking FZD4-β-catenin signalling interferes with a directional flow sensor so that cells are less able to respond to DF. We observed similar alterations to EC morphology under DF following treatment with resveratrol (Warboys et al., 2014a). Future studies should investigate whether inhibition of β-catenin signalling can switch cells exposed to atherogenic DF to an atheroprotective phenotype.

Our finding that β-catenin-dependent transcriptional activity alters cytoskeletal organisation in response to DF via WNT5A-FZD4 signalling is supported by a previous study of human coronary artery ECs cultured under static conditions. Here, gene expression profiling revealed enrichment of genes involved in cytoskeletal remodelling following exposure to WNT5A, which was associated with increased endothelial permeability (Skaria et al., 2017). The effects of WNT5A were mediated by Ryk (Skaria et al., 2017), which is consistent with our data showing a potential role for Ryk in mediating responses to DF. Although the authors did not explore whether FZD4 and β-catenin were involved, a subsequent study found that RSPO-3 was also associated with barrier disruption in ECs cultured under static conditions; in this case, downstream mechanisms were not studied (Skaria et al., 2018). Conversely, WNT5A has also been shown to stabilise the interaction of vinculin with adherens junctions and strengthen cell–cell interactions (Carvalho et al., 2019), although these experiments were performed in static ECs and WNT5A exerted its effects via Ror2, further demonstrating that Wnt-Frizzled signalling is highly context dependent. We speculate that under DF, elevated RSPO-3 and WNT5A act synergistically to increase signalling through FZD4 and Ryk, leading to increased transcriptional activity of β-catenin and expression of genes that promote the formation of stress fibres, disorganisation of adherens junctions, increased permeability and endothelial dysfunction.

In conclusion, our results demonstrate that inhibition or deletion of β-catenin reduces inflammatory signalling and enhances barrier function, pointing towards a role for β-catenin in DF-induced endothelial dysfunction. However, our previous research demonstrates that β-catenin can also play a pro-survival role in ECs exposed to DF (Tajadura et al., 2020). We have also shown that β-catenin can interact with eNOS in ECs (Warboys et al., 2014b) and that β-catenin is required for maximal activation of eNOS in ECs exposed to UF (Tajadura et al., 2020). The functions of β-catenin under flow conditions are thus complex. Further research is required to understand the dual functions of β-catenin signalling in ECs, and further understanding of upstream and downstream pathways is necessary before such signalling can form the basis of therapeutic interventions.

## MATERIALS AND METHODS

### Culture of human aortic endothelial cells and exposure to flow

Human aortic endothelial cells (HAEC) from male and female donors were obtained from PromoCell and cultured on fibronectin-coated plasticware in PromoCell Endothelial Growth Medium MV with Supplement Mix. The culture medium was replaced every 48–72 h and cells were sub-cultured using a trypsin-EDTA solution (0.25%; Sigma-Aldrich). For flow experiments, cells were seeded at passage 7 in six-well plates coated with fibronectin (10 µg ml^−1^) and cultured for 24–48 h until confluent. For immunostaining experiments, cells were seeded in glass-bottomed six-well plates (Cellvis). Once confluent, 1.902 ml fresh medium was added to each well (equivalent to 2 mm medium height). Plates were placed on an orbital shaker housed inside the incubator (Grant Instruments; 150 rpm with 5 mm orbital radius) and exposed to flow, induced by the swirling of the medium across the base of the well (Ghim et al., 2017; Warboys et al., 2019). Previous computational fluid dynamics studies have shown that ECs in the central region of the well (0–7 mm radial distance from the centre of the well) experience low-magnitude (<0.3 Pa) multidirectional flow (disturbed flow; DF), whereas ECs in the peripheral region of the well (10–16 mm radial distance from the centre of the well) experience high-magnitude (>0.5 Pa) unidirectional flow (undisturbed flow; UF) (Ghim et al., 2018; Pang et al., 2021) (see also Fig. S1). The mechanobiology of ECs exposed to flow using the method has been well characterised, with ECs in the centre of the well exhibiting endothelial dysfunction (Warboys et al., 2019).

In some experiments, cells were treated with the following inhibitors: iCRT5 (50 µM; Abcam), human DKK-1 (250 ng ml^−1^; R&D Systems) or human SFRP-1 (100 ng ml^−1^; Peprotech). Inhibitors were added for the duration of flow exposure (72 h) or for the last 24 h of flow exposure as indicated below. DMSO (0.1% v/v), PBS (0.25% v/v) or H_2_O (0.2% v/v) was used as a vehicle control, respectively. Additional details are included in Table S1.

### Computational fluid dynamics

Flow simulations were carried out with Star CCM+ (version 11.02.009, CD-adapco). A single well of a 6-well plate was represented as a cylinder with height of 10 mm and radius of 17.4 mm. The geometry was discretised using a structured cylindrical mesh with 360,000 grid elements. The explicit unsteady model was used. A no-slip condition was imposed at all walls and surface tension was neglected. The top surface of the cylinder was defined as a pressure outlet. The dynamic viscosity and density of medium were 0.78×10^3^ Pa.s and 1003 kg/m^3^, respectively. The rotation of the well was modelled by introducing a translating gravitational force with the form:

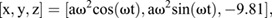
 where ‘a’ is the orbital radius of the shaker, ‘ω’ is the angular velocity and ‘t’ is time. The ‘Volume of Fluid’ model was used to track the free surface of the liquid, which had a height of 2 mm when the well was stationary. Time steps of 1×10^−4^ s were each iterated five times. Maximum wall shear stress at the base of the well was used to assess convergence. A mesh independence study was performed using 720,000 grid elements, and no difference was observed.

### Transfection with siRNA and TCF reporter plasmid

Prior to seeding into fibronectin-coated six-well plates, ECs were transfected with 100 nM MISSION predesigned validated siRNA (Sigma-Aldrich) (see Table S1) or scrambled siRNA control (Thermo Fisher Scientific) or Cignal reporter plasmid (500 ng; QIAGEN). Transfection was carried out by electroporation using a Neon Transfection System (Thermo Fisher Scientific) according to the manufacturer's instructions. Electroporated cells were seeded directly into wells containing Endothelial Growth Medium (PromoCell) (approximately 5×10^5^ cells per well) and cultured for 6–8 h until firmly adhered and confluent. The culture medium was replaced before exposure to flow (as above). For transfection experiments, ECs were exposed to flow for 48 h to ensure knockdown efficiency.

### Luciferase reporter assay

Using a template, ECs were scraped and aspirated from wells to leave only the DF or UF region, defined above. After scraping, the remaining cells were lysed with Passive Lysis Buffer (Promega) for 15 min at room temperature with constant agitation. Lysates were collected and vortexed briefly. TCF/LEF reporter activity was assessed using a Dual-Luciferase Reporter Assay System (Promega) according to the manufacturer's instructions. Luminescence of the firefly and *Renilla* luciferase reporters was quantified using a Varioskan Flash Plate Reader (Thermo Fisher Scientific). The activity of the firefly (experimental) reporter was normalised to the activity of the *Renilla* (control) reporter to account for differences in transfection efficiency. Normalised values were also corrected for protein concentration to account for any variations in cell lysis.

### Monocyte adhesion assays

THP-1 monocytes (American Type Culture Collection) were cultured in suspension in RPMI-1640 medium (Sigma-Aldrich) supplemented with 10% fetal bovine serum (FBS; Sigma-Aldrich), 2 mM L-glutamine, 100 IU ml^−1^ penicillin-streptomycin and 50 µM β-mercaptoethanol. The culture medium was replaced every 48–72 h and cell density maintained at 8×10^5^–1×10^6^ cells ml^−1^. Prior to adhesion assays, THP-1 monocytes were incubated with 1 µg ml^−1^ Calcein-AM (Thermo Fisher Scientific) for 20 min at 37°C. Following labelling, monocytes were washed, re-suspended in EC growth medium and added to endothelial monolayers that had been exposed to flow for 72 h in the presence or absence of iCRT5. In a subset of experiments, ECs were pre-treated with 10 ng/ml TNFα for the final 24 h of flow exposure. THP-1 monocytes (1×10^6^ per well) were incubated with EC monolayers at 37°C under static conditions for 60 min. Unbound monocytes were removed by extensive washing and adherent cells fixed using 4% paraformaldehyde for 5 min. Images of adherent monocytes were acquired with a Zeiss Axioplan epifluorescence microscope using a 20× objective with 470/40 nm excitation and 525/50 nm emission filters. The total number of adherent monocytes was captured across five fields of view from the centre of each well.

### RNA isolation and qRT-PCR

Using a template, ECs were scraped and aspirated from wells to leave only the DF or UF region (as above), from which RNA was obtained by addition of RLT lysis buffer (QIAGEN). In order to obtain enough material, cells from DF or UF regions from three wells were pooled. RNA was isolated using RNeasy Mini Kits with on-column DNase digestion (QIAGEN) according to the manufacturer's instructions. A High-Capacity Reverse Transcription Kit (Thermo Fisher Scientific) was used to prepare cDNA according to the manufacturer's instructions. Transcript levels were assessed by quantitative real-time PCR (qRT-PCR) (Applied Biosystems StepOnePlus Real-Time PCR System) using Fast SYBR Green MasterMix (Applied Biosystems) and gene-specific primers (see Table S2). Reactions were carried out using an Applied Biosystems StepOnePlus Real-Time PCR System as follows: 95°C for 20 s, followed by 40 cycles of 95°C for 3 s and 60°C for 30 s. All reactions were performed in triplicate and relative expression assessed by the ΔΔCt method, using *GAPDH* as a reference gene.

### Protein extraction and western blotting

Using a template, ECs were scraped and aspirated from wells to leave only the DF or UF region (as above), from which protein lysates were obtained by addition of RIPA buffer (0.1% SDS, 1% Triton-X 100, 0.5% sodium deoxycholate, 150 mM NaCl and 50 mM Tris-HCl at pH 7.4) supplemented with protease and phosphatase inhibitor cocktails (Sigma-Aldrich). In order to obtain enough material, cells from DF or UF regions from three wells were pooled. Lysates were incubated on ice for 45 min with regular vortexing and centrifuged at 13,845 ***g*** for 10 min to separate soluble and insoluble fractions. For analysis of cytosolic and nuclear fractions, cells were lysed using NE-PER Nuclear and Cytoplasmic Extraction Reagent (Thermo Fisher Scientific) according to the manufacturer's instructions. Cells were pooled from DF or UF regions from six wells to obtain enough material. Lysates were analysed by SDS-PAGE and immunoblotting. Membranes were incubated with primary antibodies (see Table S1) overnight at 4°C and visualised with HRP-conjugated anti-IgG secondary antibodies (see Table S1) and enhanced chemiluminescence substrates (Bio-Rad). The expression of PDHX or calnexin was used as a loading control. For transparency, representative blots are included in Fig. S9.

### Immunofluorescence staining

ECs were fixed in 4% paraformaldehyde for 20 min, permeabilised with 0.1% Triton X-100 for 3 min and blocked with 5% bovine serum albumin for 1 h before incubating with primary antibodies overnight at 4°C (see Table S1). Immunostaining was visualised using relevant Alexa Flour 488- or 568-conjugated secondary antibodies (see Table S1). In a subset of experiments, ECs were incubated with Alexa Fluor 488–Phalloidin (Thermo Fisher Scientific) for 20 min at room temperature to visualise the actin cytoskeleton. Nuclei were stained by incubation with 5 µM DRAQ5. ECs were imaged directly in the well using a Leica SP5 or SP8 Laser Scanning Confocal Microscope with excitation at 488, 565 and 633 nm. A minimum of four fields of view were studied for each flow condition in each well using identical laser power and detector gain settings. Cell elongation was quantified by calculating the length-to-width ratio. ZO-1 expression within cell junctions was quantified by measuring mean fluorescence intensity within standardised regions of interest spanning cell junctions.

### Quantification of EC permeability

ECs were seeded in six-well plates coated with biotinylated gelatin (Neta Scientific) (Dubrovskyi et al., 2013) and cultured for 72 h under static conditions followed by exposure to flow for 72 h using the orbital shaker. The medium was replaced with reduced serum (2.5% FBS) EC growth medium (PromoCell) for the final 24 h of flow exposure. FITC–avidin (Thermo Fisher Scientific) diluted in reduced serum medium (0.38 µM) was added to wells, which were then incubated at 37°C for 3 min. Binding of fluorescently labelled avidin to biotin occurs underneath ECs, in particular at the intercellular junctions (Ghim et al., 2017), and can be imaged. Monolayers were washed with PBS to remove unbound tracer before fixing with 4% paraformaldehyde for 10 min. Fixed monolayers were then incubated with an anti-VE-cadherin antibody to delineate cell junctions. Wells were imaged with a Leica SP5 inverted confocal microscope using a 10×, 0.40 NA objective. Nine *z*-stack images were taken from the DF region in the centre of the well (3×3 tile scan) and 12 images from the UF regions at the edge of the well. For quantification of FITC–avidin accumulation, a maximum-projection image was computed for each stack of images and the tracer fluorescence was distinguished from background noise by intensity and area thresholding. The resulting binarised image was overlaid on the original image and used as a mask to quantify the FITC–avidin accumulation.

### Immunostaining and imaging of porcine aortas

Aortic arches were obtained from juvenile pigs (<16 weeks of age) culled at Newman's abattoir (Farnborough, Surrey, UK) or from The Pirbright Institute (Pirbright, UK). Tissue obtained from The Pirbright Institute was collected in accordance with Directive 2010/63/EU of the European Parliament on the protection of animals used for scientific purposes, under authorisation of the UK Home Office (Project License No. 70/8852) and the Animal Welfare and Ethical Review Board of The Pirbright Institute (Matos et al., 2022). Pigs were euthanised by overdose of 10 ml pentobarbital (Dolethal, 200 mg/ml solution for injection, Vetoquinol UK) (Matos et al., 2022). The aortic arch was excised and fixed immediately in 4% paraformaldehyde for up to 7 days. Aortas were dissected into 1 cm^2^ sections from either the inner or outer curvature (exposed to DF or UF, respectively) according to previous computational fluid dynamic analysis (Serbanovic-Canic et al., 2017), and the sections were placed into a glass-bottomed 24-well plate. The tissue was permeabilised with Triton X-100 (0.1% v/v) for 5 min before blocking with normal goat serum (10% v/v) for 1 h. It was then stained with anti-β-catenin and anti-FZD4 antibodies (see Table S1) overnight at 4°C for 24 h and 48 h, respectively. Immunostaining was visualised using relevant Alexa Flour 488- or 568-conjugated secondary antibodies. Nuclei were stained using DAPI (1 µg ml^−1^) and tissue was imaged *en face* using a Leica SP8 confocal microscope with excitation at 405, 488 and 561 nm. A minimum of four fields of view were studied for each flow condition using identical laser power and detector gain settings.

### Statistical analysis

All data are presented as mean±standard error of the mean (s.e.m.). A minimum of three independent experiments was carried out for each investigation. Cells from at least three independent donors were used for each experiment. Power calculations were carried out using GraphPad StatMate to ensure 80% power. Data were analysed using GraphPad Prism v9.00. A Shapiro–Wilk normality test was used to determine whether data were normally distributed. For normally distributed data, statistical significance was determined using a Student's two-tailed paired *t*-test when comparing two conditions or by one-way or two-way ANOVA with Tukey's multiple comparison test when comparing multiple conditions. Where normalised data are presented, statistical analysis was carried out using a Mann–Whitney test to compare two conditions or Kruskal–Wallis analysis with Dunn's multiple comparison test to compare multiple conditions. For all data, *P<0.05, **P<0.01, ***P<0.001, ****P<0.0001.

## Supplementary Material

10.1242/joces.260449_sup1 Supplementary information
