# Supplementary material for: Disturbed flow increases endothelial inflammation and permeability via a Frizzled-4-β-catenin-dependent pathway
Source: J Cell Sci. 2023 Mar 24;136(6):jcs260449. doi: 10.1242/jcs.260449 (PMC10112981; doi:10.1242/jcs.260449)
Supplement: Supplementary information [file joces-136-260449-s1.pdf]

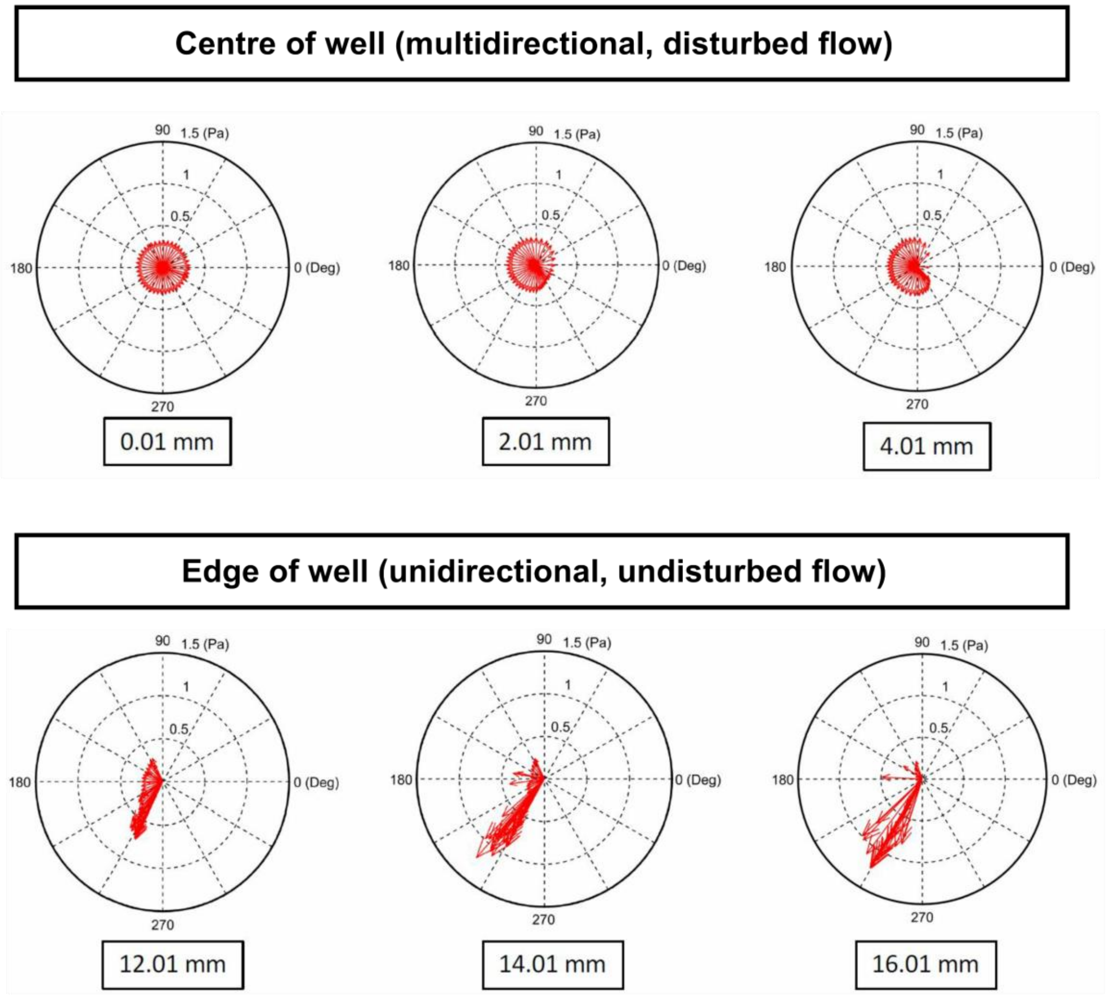

Fig. S1. Polar plots showing direction of WSS vectors in swirling wells

Individual polar plots of the magnitude and direction of instantaneous WSS vectors during one cycle. Each plot applies to one radial distance from the centre of the well. Each arrow represents an instantaneous WSS vector, whose length represents its magnitude, which is plotted at angles corresponding to the shear direction.

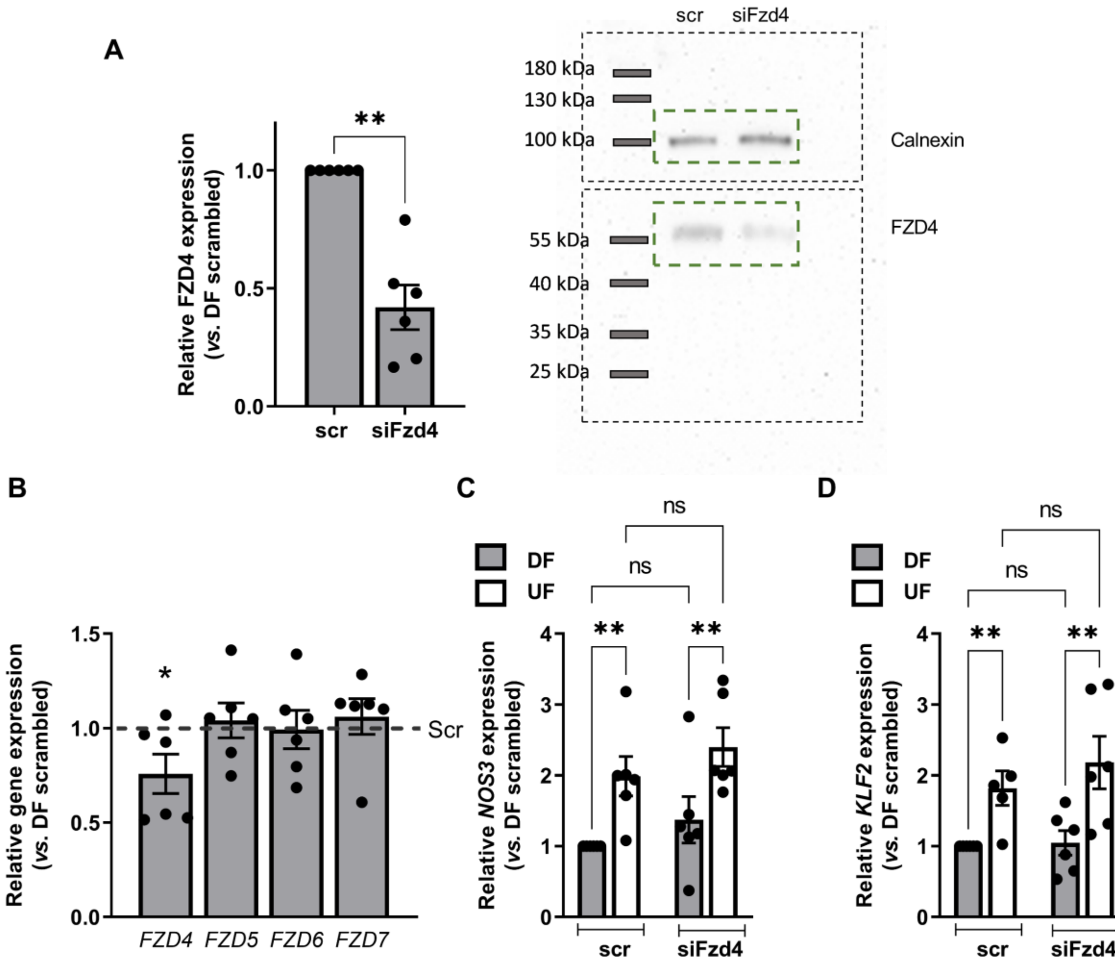

Fig. S2. Efficacy and specificity of Frizzled-4 knockdown using siRNA

(A-D) HAEC were exposed to flow for 48h following transfection with Frizzled-4 siRNA or scrambled controls. **(A)** Protein lysates were obtained from HAEC exposed to DF and the expression of Frizzled-4 assessed by western blot using calnexin as a loading control (n=6; analysis by Mann-Whitney test). **(B)** RNA was harvested from EC exposed to DF and the expression of Frizzled-4, Frizzled-5, Frizzled-6 and Frizzled-7 was determined by qRT-PCR using *GAPDH* as a housekeeping gene (n=6; analysis by Mann-Whitney test; results shown relative to DF scrambled control (dashed line)). **(C-D)** RNA was harvested from EC exposed to DF and UF and the expression of **(C)** *KLF-2* and **(D)** *NOS3* was determined by qRT-PCR using *GAPDH* as a housekeeping gene (n=6; analysis by two-way ANOVA with Tukey's multiple comparison test; results shown relative to DF scrambled control).

Journal of Cell Science • Supplementary information

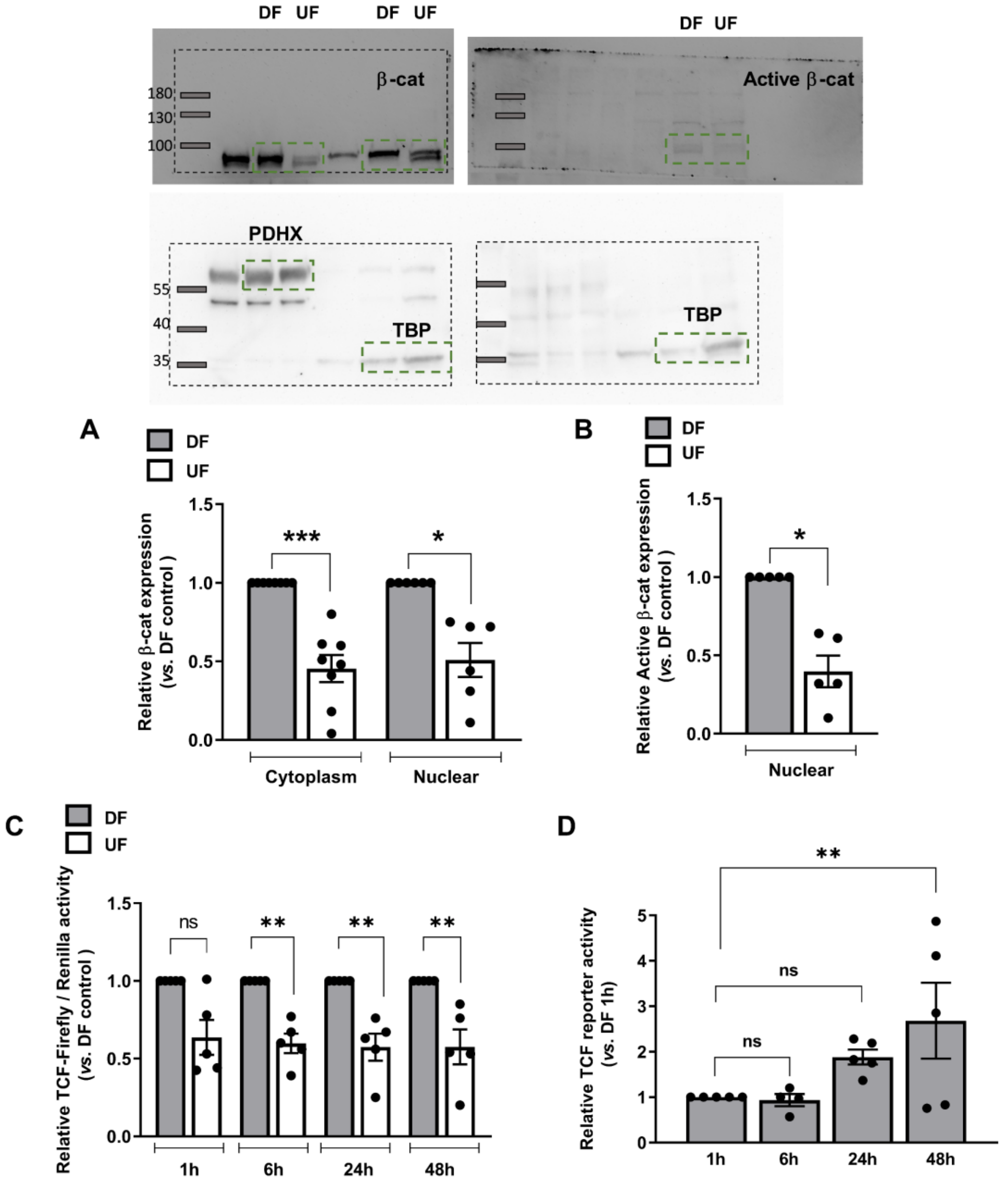

Fig. S3. Disturbed flow increases the expression and activation of β-catenin in human aortic endothelial cells

(A-B) HAEC were exposed to flow for 72h. Lysates were prepared from EC exposed to DF and UF and subject to sub-cellular fractionation. Cytosolic and nuclear fractions were analysed by western blot using **(A)** total β-catenin (n=6-8) and **(B)** active β-catenin antibodies (n=5). PDHX and TBP were used as loading controls for cytosolic and nuclear fractions respectively; analysis by Mann Whitney test; representative blots shown above. **(C)** HAEC were transfected with Cignal TCF/LEF reporter construct prior to flow exposure for 1h – 48h. Lysates were prepared from cells exposed to DF or UF and *Firefly* and *Renilla* luciferase activity was recorded. Ratios were corrected for protein content of lysates. **(C)** Results shown relative to DF control at each time point and analysed by Mann-Whitney test at each time point (n=5). **(D)** Results shown relative to DF 1h and analysed by one-way ANOVA (n=5).

Journal of Cell Science • Supplementary information

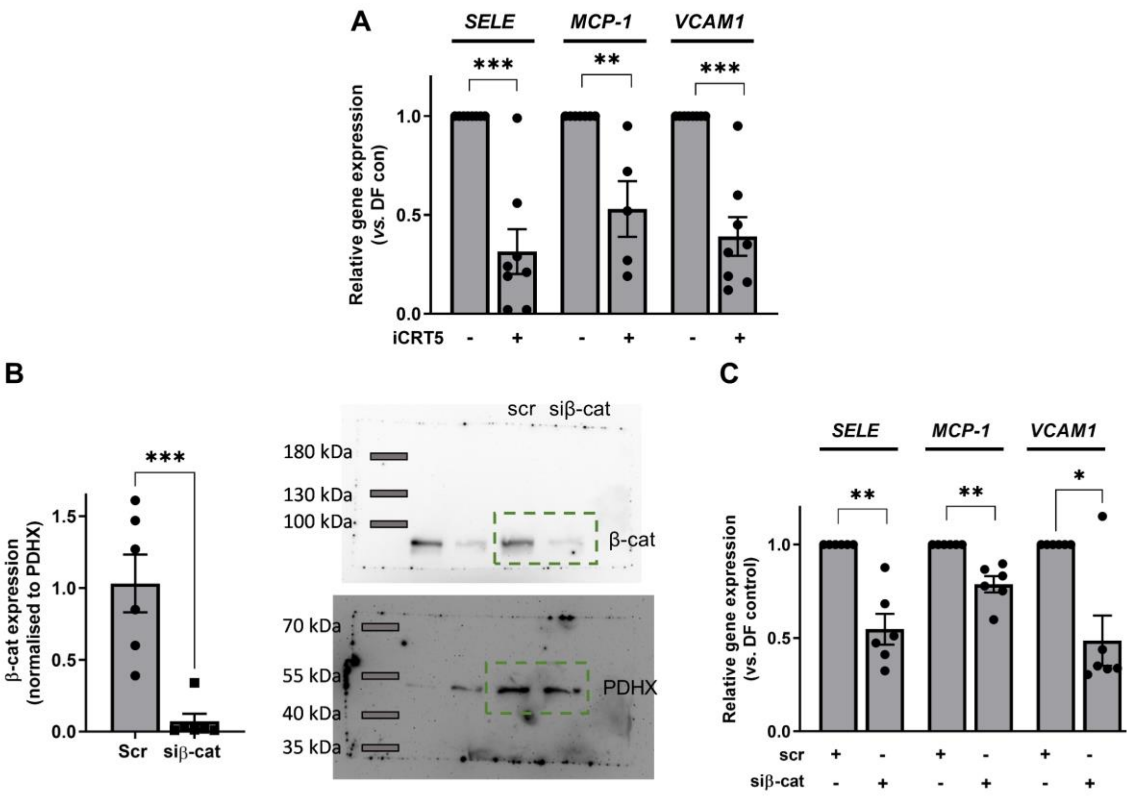

Fig. S4. Expression of pro-inflammatory genes in EC exposed to disturbed flow following inhibition or knockdown of β-catenin

(A) HAEC were exposed to flow for 72h with iCRT5 (50 μM) added for the duration of flow exposure (72h). RNA was harvested from EC exposed to DF and the expression of *E-SEL, MCP-1 and VCAM1* determined by qRT-PCR using *GAPDH* as a housekeeping gene (n=5-8; analysis by Mann-Whitney test). **(B-C)** HAEC were exposed to flow for 72h following transfection with β-catenin siRNA (siβ-cat). **(B)** The expression of β-catenin protein was assessed by western blot using PDHX as a loading control (n=6; analysis by analysis by unpaired t-test; representative blot shown next to the graph). **(C)** RNA was harvested from EC exposed to DF and the expression of pro-inflammatory genes assessed by qRT-PCR using *GAPDH* as a housekeeping gene (n=6; analysis by Mann-Whitney test).

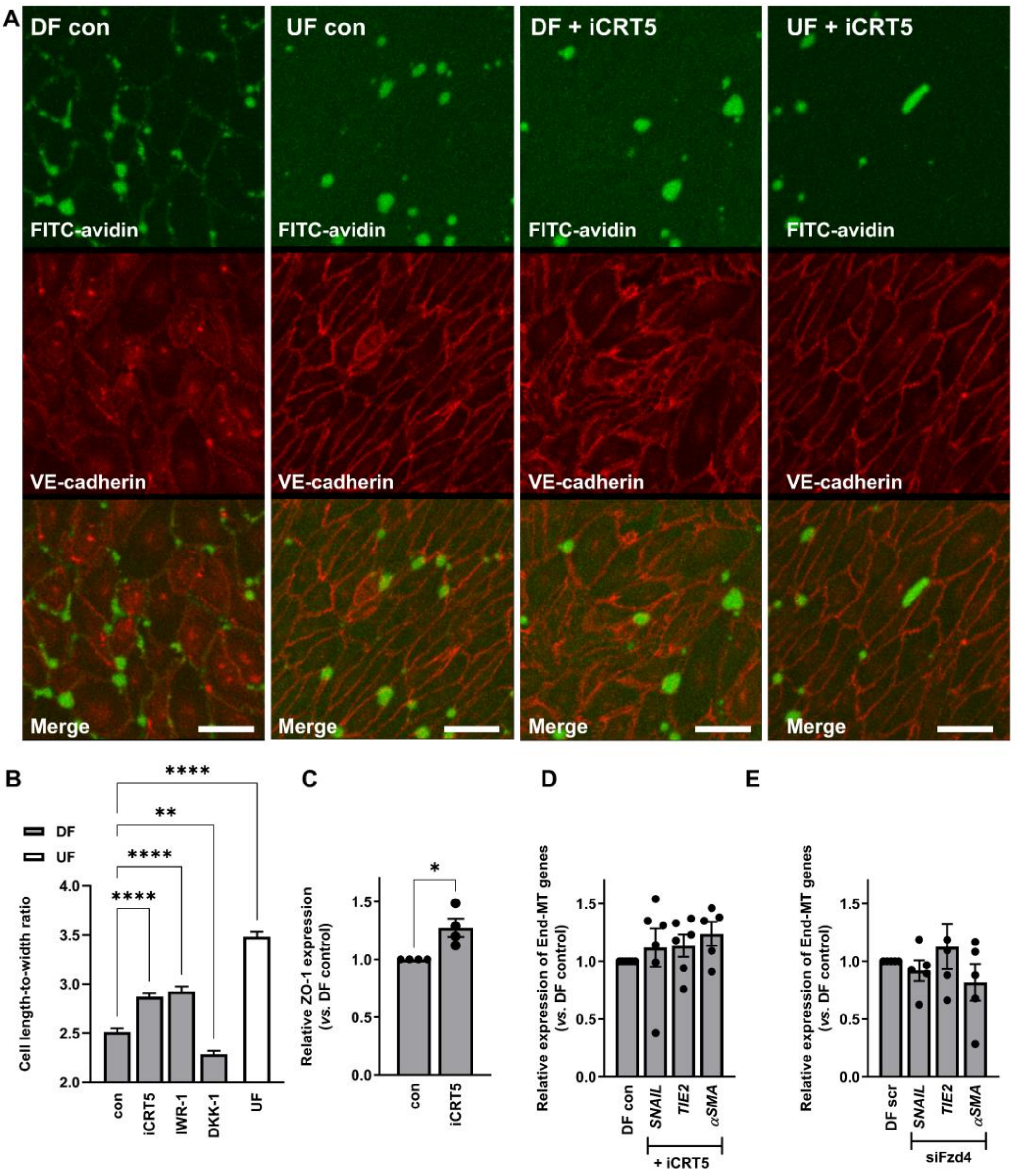

Fig. S5. Permeability and morphology of HAEC

(A) HAEC were cultured on biotinylated-gelatin and exposed to flow for 72h then treated with iCRT5 (50μM) for the last 24h of flow exposure. FITC-avidin was added to monolayers immediately after flow cessation. Images show areas where FITC-avidin binds to biotinylated-gelatin underlying EC. Cells were counterstained with an anti-VE-cadherin antibody (images shown are maximum projections of z-stacks; scale = 50μm). **(B)** HAEC were fixed and stained with VE-cadherin and DRAQ5 and viewed at x10 magnification. The length-to-width ratio of cells in at least 3 fields of view per experiment (approx. 200 cells per field) was determined in cells exposed to DF and treated with iCRT5 (50 μM), IWR-1 (10 μM) or DKK-1 (250 ng.ml^{-1}). The length-to-width ratio of EC exposed to UF are shown for comparison (n=3; analysis by Kruskal-Wallis test with uncorrected Dunn's test). (**C**) HAEC were exposed to flow for 72h and treated with iCRT5 (50μM) for the last 24h of flow exposure. HAEC were fixed and stained with ZO-1 and DRAQ5. Mean fluorescence intensity of standardised regions of interest spanning cell junctions was used to quantify ZO-1 expression (n=4; analysis by Mann-Whitney test) **(D)** HAEC were exposed to flow for 72h and treated with iCRT5 (50μM) for the last 24h of flow exposure or **(E)** transfected with Frizzled-4 siRNA (siFzd4) and exposed to flow for 48h. RNA was harvested from EC exposed to DF and expression of *TIE2*, α-smooth muscle actin (*α-SMA*) and *SNAIL* assessed by qRT-PCR using *GAPDH* as a housekeeping gene (n=5-6; analysis by Mann-Whitney test).

Journal of Cell Science • Supplementary information

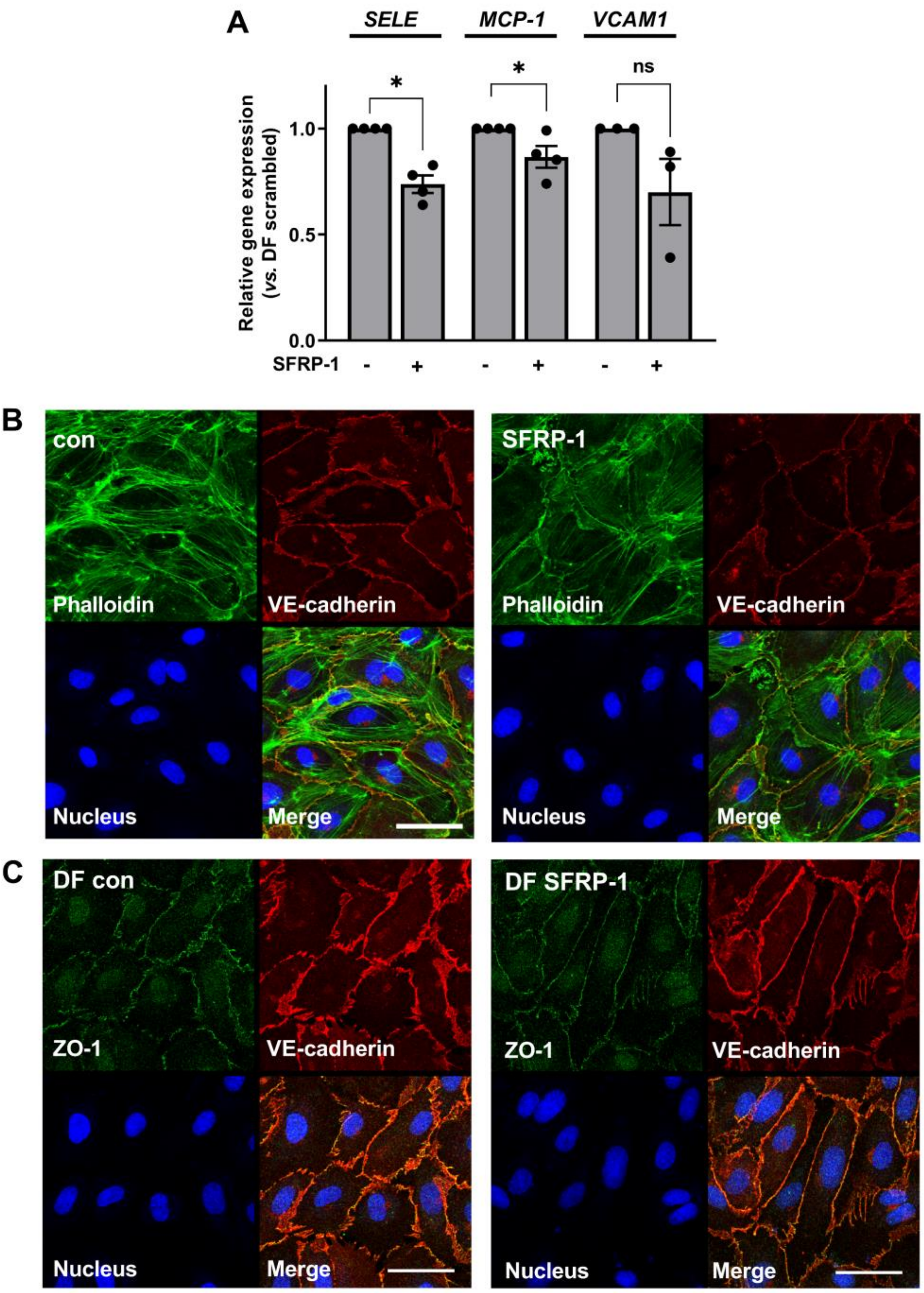

Fig. S6. SFRP-1 reduces inflammatory signalling in EC exposed to disturbed flow and alters cytoskeletal organisation

(A-C) HAEC were exposed to flow for 72h and treated with SFRP-1 (200 ng.ml^{-1}) for the last 24h of flow exposure. **(A)** RNA was harvested from EC exposed to DF and the expression of pro-inflammatory genes assessed by qRT-PCR using GAPDH as a housekeeping gene (n=3-4; analysis by Mann-Whitney test). **(B-C)** EC were fixed and incubated with anti-VE-cadherin antibody, 488-phalloidin or anti-ZO-1 antibody and DRAQ5 nuclear stain (n=4; scale = 50 μm; representative images shown).

Journal of Cell Science • Supplementary information

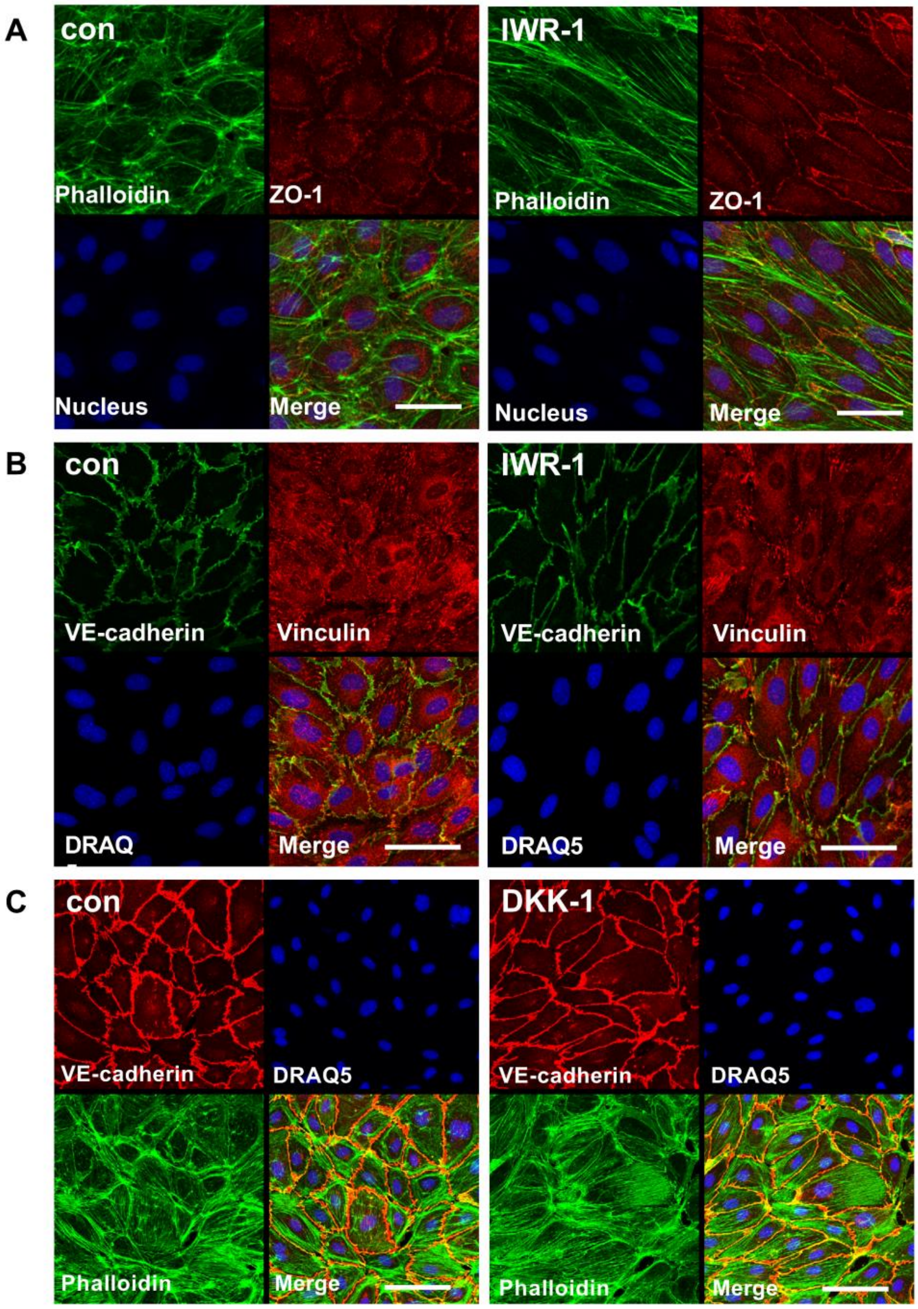

Fig. S7. IWR-1 but not DKK-1 alters cytoskeletal organisation in EC exposed to disturbed flow

(A-C) HAEC were exposed to flow for 72h and treated with **(A-B)** IWR-1 (10µM) or **(C)** DKK-1 (250 ng.ml^{-1}) for the final 24h of flow exposure. **(A-C)** EC were fixed and stained with DRAQ5 nuclear stain and **(A)** 488-Phalloidin and anti-ZO-1 antibody or **(B)** VE-cadherin and vinculin antibodies or **(C)** 488-Phalloidin and anti-VE-cadherin antibody. Images obtained from 4 fields of view (n=5; representative images shown; scale = 50µm).

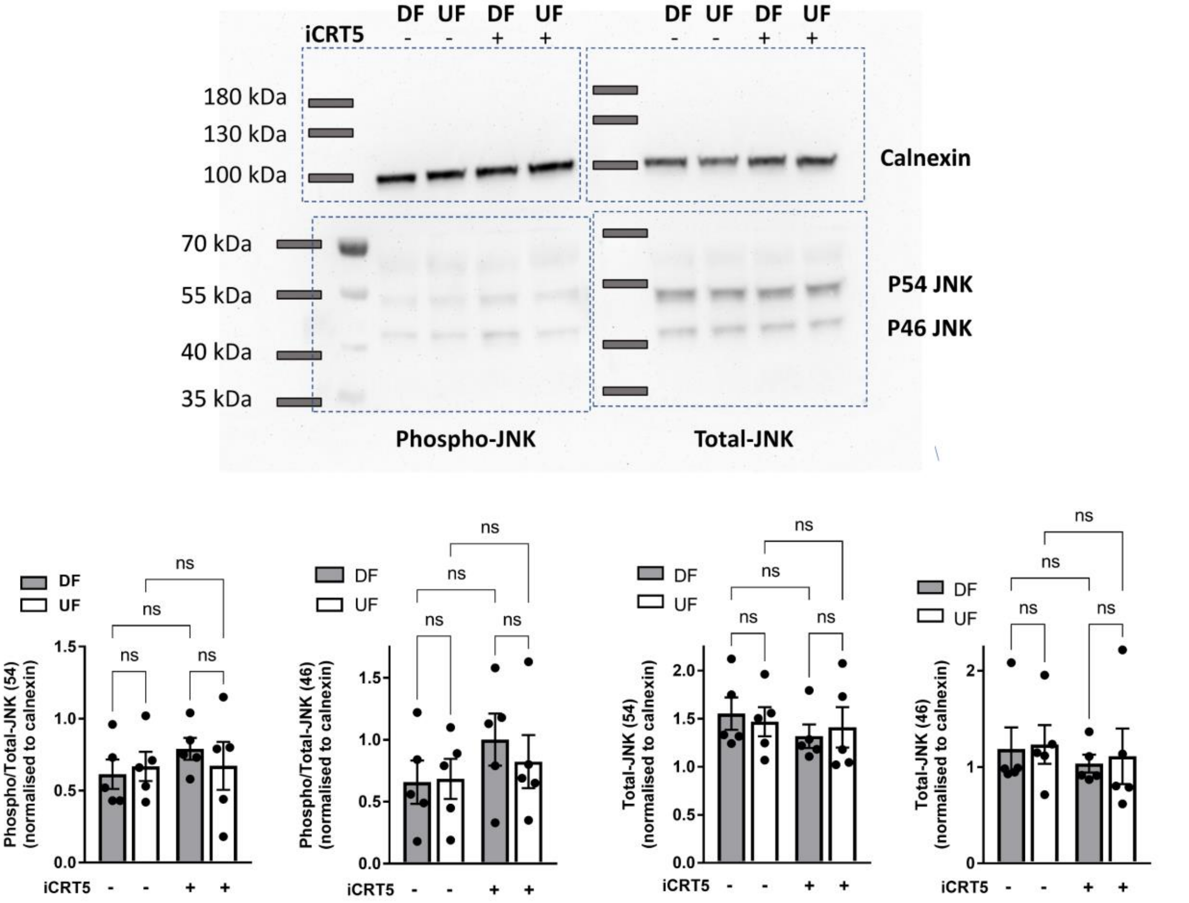

Fig. S8. Expression and phosphorylation of JNK in EC exposed to flow

HAEC were exposed to flow for 72h and treated with iCRT5 (50µM) for the last 24h of flow exposure. Lysates from EC exposed to DF and UF were assessed by western blot using phospho-JNK and total JNK antibodies. Calnexin was used as a loading control. Results analysed by one-way ANOVA (n=5; representative blots shown above).

Journal of Cell Science • Supplementary information

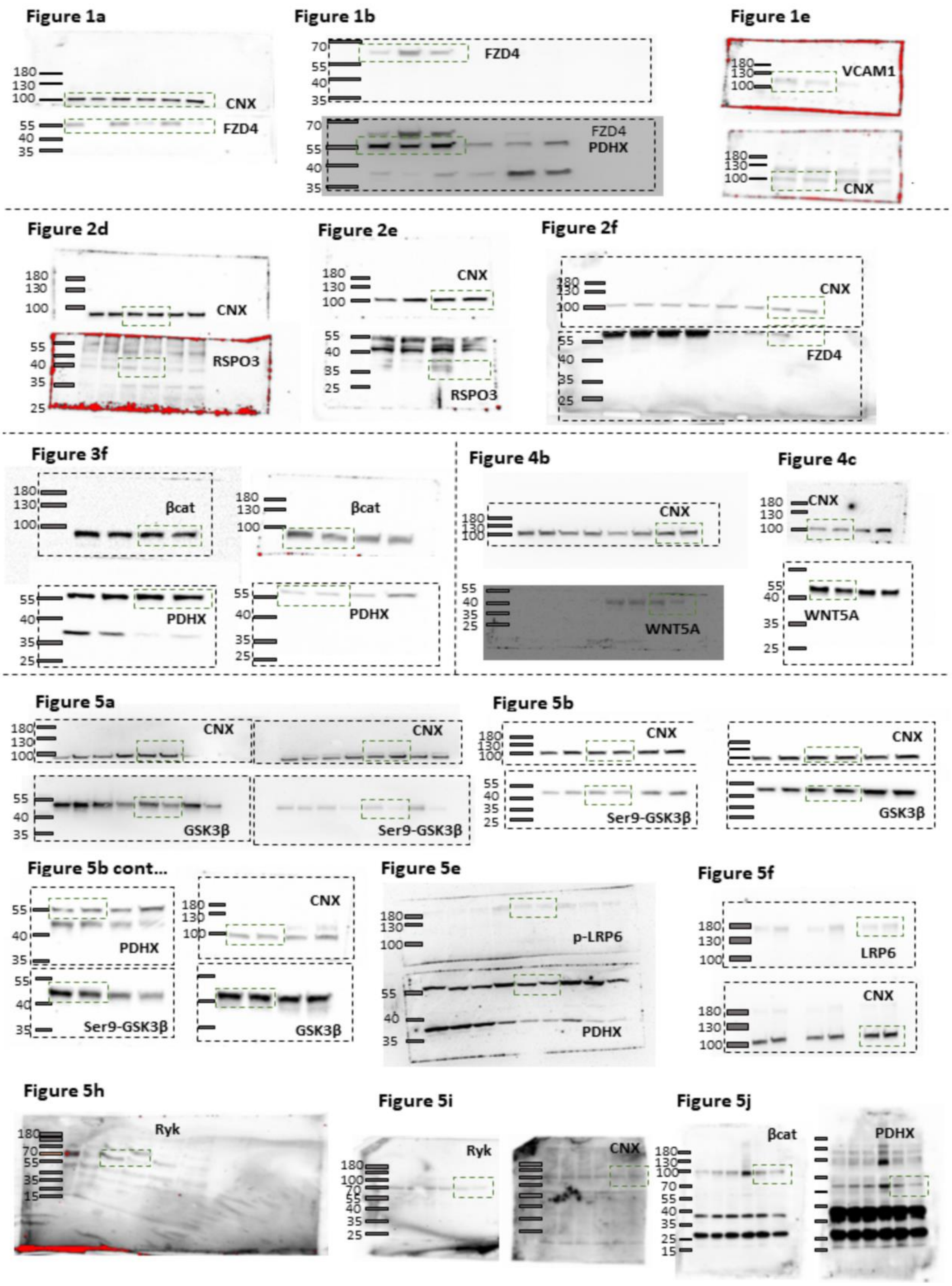

Fig. S9. Uncropped images of western blots used throughout the manuscript. Dotted lines denote bands featured in the relevant figures.

Journal of Cell Science • Supplementary information

Table S1. Details of antibodies, reagents and cells used

Antibody target	Species	Source	Catalog #	Application	Dilution
β-catenin	Mouse	BD Biosciences	610153	IF	1 in 100
β-catenin	Rabbit	Cell Signaling Technology	9582	WB	1 in 3000
β-catenin (active)	Rabbit	Millipore	05-665	WB	1 in 1000
Calnexin	Goat	Santa Cruz	sc-6465	WB	1 in 3000
Frizzled-4	Goat	Santa Cruz	sc-66450	WB	1 in 1000
Frizzled-4	Rabbit	ThermoFisher	710731	IF	1 in 100
GSK3β	Rabbit	Cell Signaling Technology	9315	WB	1 in 1000
GSK3β(Ser9)	Rabbit	Cell Signaling Technology	9322	WB	1 in 1000
JNK	Rabbit	Cell Signaling Technology	9252	WB	1 in 1000
JNK (Thr183/Tyr185)	Rabbit	Cell Signaling Technology	4668	WB	1 in 1000
Lrp6	Rabbit	Cell Signaling Technology	3395	WB	1 in 1000
Lrp6(Ser1490)	Rabbit	Cell Signaling Technology	2568	WB	1 in 1000
PDHX (E3BP)	Mouse	Santa Cruz	sc-377255	WB	1 in 1000
RSPO-3	Rabbit	Proteintech	17193-1-AP	WB	1 in 1000
Ryk	Mouse	R&D Systems	MAB4907	WB	1 in 1000
TBP	Rabbit	Cell Signaling Technology	44059	WB	1 in 2000
VCAM-1	Rabbit	Cell Signaling Technology	13662	WB	1 in 1000
VE-cadherin	Rabbit	Cell Signaling Technology	2500	IF	1 in 500
VE-cadherin	Mouse	BD Biosciences	555661	IF	1 in 500
Vinculin	Mouse	Sigma	V4505	IF	1 in 500
WNT5A	Rabbit	Proteintech	55184-1-AP	WB	1 in 1000
ZO-1	Rabbit	Cell Signaling Technology	13663	IF	1 in 250
**Secondary antibodies**					
Alexa Fluor-488 (rabbit)	Goat	Thermo Fisher Scientific	A11008	IF	1 in 500
Alexa Fluor-568 (rabbit)	Goat	Thermo Fisher Scientific	A11011	IF	1 in 500
Alexa Fluor-568 (mouse)	Goat	Thermo Fisher Scientific	A11004	IF	1 in 500
HRP anti-goat IgG	Donkey	Abcam	ab6885	WB	1 in 10000
HRP anti-mouse IgG	Goat	Abcam	ab205719	WB	1 in 10000
HRP anti-rabbit IgG	Goat	Cell Signaling Technology	7074S	WB	1 in 10000

Reagent	Species	Source	Catalog #	Dilution	Vehicle control (v/v)
488-Phalloidin	na	ThermoFisher	A12379	1 in 40	na
DRAQ5	na	Biostatus	DR50200	5 µM	na
DKK-1	Human	R&D Systems	5439-DK-010	250 ng/ml	PBS (0.1%)
iCRT5	na	Abcam	ab142141	50 µM	DMSO (0.1%)
IWR-1	na	Sigma-Aldrich	I0161	10µM	DMSO (0.1%)
SFRP-1	Human	PeproTech	120-29	200 ng/ml	PBS (0.1%)
TNFα	Human	R&D Systems	210-TA-020	10ng/ml	PBS (0.1%)

Journal of Cell Science • Supplementary information

Cell type	Species	Source	Catalog #
Human aortic endothelial cells	Human	Promocell	C-12271
THP-1 monocytes	Human	ATCC	TIB-202

siRNA	Species	Source	Catalog # / ID
β-catenin siRNA (siβcat)	Human	Sigma	Predesigned siRNA ID: SASI_Hs01_00117960
Frizzled-4 siRNA (siFzd4)	Human	Sigma	Predesigned siRNA ID: SASI_Hs01_00241018
RSPO-3 siRNA (siRSPO-3)	Human	Sigma	Predesigned siRNA ID: SASI_Hs01_00012894
Ryk siRNA (siRyk)	Human	Sigma	Predesigned siRNA ID: SASI_Hs01_00047742
scrambled siRNA (scr)	Human	ThermoFisher	Silencer Select Negative Control #4390846
Wnt5a siRNA (siWnt5a)	Human	Sigma	Predesigned siRNA ID: SASI_Hs01_00202618

Journal of Cell Science • Supplementary information

Table S2. Details of primers used for qRT-PCR

Gene	Forward	Reverse
CTNNB1	TGCCCTGGCTATGTGAGTTT	TCAAATACCCTGCATAGTACGCT
CLAUDIN-5	CCTGTGCCACCGCTTTTTG	CAGCACTGTCTCTCTCATCCC
ENOS	CATCTTCAGCCCCAAACGGA	AGCGGATTGTAGCCTGGAAC
ESAM	TCACCAACCTTTCGTCTTCCA	CCAGCGTCACATTACATTGGG
E-SEL	GCTCTGCAGCTCGGACAT	GAAAGTCCAGCTACCAAGGGAAT
FZD4	GGATGCTCTGTGGCCTTTCT	GGGCATGTGTAGCAGGAAGT
FZD5	GAGAGACGGTTAGGGCTCG	TTCTCAGCGGAGTGACCC
FZD6	GGGAACGGTGGGTTAGACG	CTGGGTCAATTACTCGGGGG
FZD7	GTCGTGTTTCATGATGGTGC	CGCCTCTGTTCGTCTACCTC
GAPDH	CTATAAATTGAGCCCGCAGCC	ACCAAATCCGTTGACTCCGA
JAM-A	ACGGGAAGACACTGGGACATA	GGATGGAGGCACAAGCAC
JAM-B	AGCAGTAGAGTACCAAGAGGCTA	CTCCGACCCAGTTTCTTCCA
JAM-C	CAAAATTCAGGGAGACTTGGCG	CCTCACAGCGATAAAGGGCT
KLF2	TGGGCATTTTTGGGCTACCT	CCCAGTTCCAAGCAACCAGA
MCP-1	AGGTGACTGGGGCATTGAT	GCCTCCAGCATGAAAGTCTC
PECAM-1	CACAGATGAGAACCACGCCT	GGCCCCTCAGAAGACAACAT
RNF43	TTGTTTCACCCCCGTGGATT	TCACTTGGCATTGCTCTTCC
RSPO1	GCTGGCAAGGACTGGTGTTT	TGGTTGATTGCCTCGACACC
RSPO2	AACCGATGGAGACGCAGTAA	GTTGACATCGGCTACACCCA
RSPO3	GTAGGGGAGAAAGCCACCAC	ACCAGGTTCTCTGAGTTAGCA
RSPO4	CACAATGGAAAGACCTGCGG	GACTCAGAAAGCACCTGGCA
**αSMA**	CCGGGACTAAGACGGGAATC	TTGTCACACACCAAGGCAGT
SNAIL	CGAGTGGTTCTTCTGCGCTA	TGCAGCTCGCTGTAGTTAGG
TIE2	TCCTTGGCTCTGCTGGAATG	GGTTCTTCCCTCACGTTTTGG
VCAM-1	GTCTCCAATCTGAGCAGCAA	TGGGAAAAACAGAAAAGAGGTG
VE-CADHERIN	ATGAGATCGTGGTGGAAGCG	TGTGTACTTGGTCTGGGTGAAG
ZO-1	GTGGGTAACGCCATCCTCTG	CCATTGCTGTGCTAGTGAGC
ZNRF3	CTGGGTAGGAGTGGTGAAGC	CTTGCCTAGGACAGTGAGGC

Journal of Cell Science • Supplementary information
