# Supplementary material for: Disturbed flow increases endothelial inflammation and permeability via a Frizzled-4-β-catenin-dependent pathway
Source: J Cell Sci. 2023 Mar 24;136(6):jcs260449. doi: 10.1242/jcs.260449 (PMC10112981; doi:10.1242/jcs.260449)
Supplement: Supplementary file 1 [file joces-136-260449_review_history.pdf]

Journal of Cell Science

Disturbed flow increases endothelial inflammation and permeability via a Frizzled-4-ß-catenin-dependent pathway

Matthew Rickman, Mean Ghim, Kuin Pang, Ana Cristina von Huelsen Rocha, Elena M. Drudi, Macià Sureda-Vives, Nicolas Ayoub, Virginia Tajadura-Ortega, Sarah J. George, Peter D. Weinberg and Christina M. Warboys
DOI: 10.1242/jcs.260449

Editor: Andrew Ewald

Review timeline

Original submission:	25 July 2022
Editorial decision:	30 August 2022
First revision received:	16 January 2023
Editorial decision:	14 February 2023
Second revision received:	15 February 2023
Accepted:	19 February 2023

Original submission

First decision letter

MS ID#: JOCES/2022/260449

MS TITLE: Frizzled-4 regulates ß-catenin in endothelium exposed to disturbed flow causing proinflammatory activation and increased permeability

AUTHORS: Matthew Rickman, Mean Ghim, Kuin Pang, Ana Cristina von Huelsen-Rocha, Elena M Drudi, Macià Sureda-Vives, Nicolas Ayoub, Virginia Tajadura-Ortega, Sarah J George, Peter D Weinberg, and Christina M Warboys
ARTICLE TYPE: Research Article

We have now reached a decision on the above manuscript.

To see the reviewers' reports and a copy of this decision letter, please go to: https://submit-jcs.biologists.org and click on the 'Manuscripts with Decisions' queue in the Author Area. (Corresponding author only has access to reviews.)

As you will see, all three reviewers share enthusiasm for the study and its conclusions but raise a number of substantial criticisms that prevent me from accepting the paper at this stage. They suggest, however, that a revised version might prove acceptable, if you can address their concerns. If you think that you can deal satisfactorily with the criticisms on revision, I would be pleased to see a revised manuscript. We would then return it to the reviewers.

Please ensure that you clearly highlight all changes made in the revised manuscript. Please avoid using 'Tracked changes' in Word files as these are lost in PDF conversion.

I should be grateful if you would also provide a point-by-point response detailing how you have dealt with the points raised by the reviewers in the 'Response to Reviewers' box. Please attend to all of the reviewers' comments. If you do not agree with any of their criticisms or suggestions please explain clearly why this is so.

© 2023. Published by The Company of Biologists under the terms of the Creative Commons Attribution License (https://creativecommons.org/licenses/by/4.0/).

Reviewer 1

Advance summary and potential significance to field

This manuscript by Rickman and colleagues investigates the role of Frizzled-4/beta-catenin pathway in regulating endothelial cell dysfunction in response to disturbed flow. The authors find that FZD4 expression is induced by disturbed flow in human aortic cells and mouse aortas and identify an atypical FZD4/beta-catenin pathway which regulates endothelial inflammatory activation and permeability under disturbed flow conditions. The study presents novel findings and provides mechanistic insights into responses of endothelial cells to flow which are known to be complex and also highly relevant for the development of cardiovascular disease.
Overall, this manuscript is interesting, well-written and insightful. The experiments are well-designed and executed and the majority of conclusions are justified by the data presented. However, to be suitable for publication, the authors should address the comments listed below.

Comments for the author

Major:
1) To better understand the mechanism of FZD4 regulation by flow, the authors should determine whether disturbed flow induces FZD4 expression, or whether undisturbed flow suppresses its expression by testing FZD4 expression in static conditions.

2) The authors should quantify FZD4 expression in the mouse aorta (Figure 1B, en face staining). Were any negative controls used for this staining (for example isotype-matched controls or omission of primary antibody)? I have some doubts about the specificity of this staining, since a strong nuclear signal is observed (indicated by purple colour in the merged images of FZD4 (red) and DAPI (blue)), suggesting nuclear localisation of FZD4 – is this expected?

3) The authors apply unpaired or paired t-tests to compare 2 groups where one group (the control) is normalised to 1. T-tests are parametric tests and the assumptions are that the data from each group is normally distributed and that the variance within each group is similar, therefore using a t-test when the values in one group are 1,1,1,1… etc seems inappropriate. A non-parametric test would need to be used in this case or non-normalised data should be used to conduct a t-test.

4) What are the area-averaged values of wall shear stress magnitude for the DF and UF regions in this model?

5) When the authors knock down FZD4 using siRNA (Supplementary Figure 2B), in half of the samples they observe a decrease in FZD4 mRNA expression that is <20% and in one case even an increase in FZD4 mRNA expression in the cells transfected with FZD4 siRNA compared to controls. This seems like an insufficient level of knockdown and doesn't fit FZD4 protein data, where in 5 out of 6 experiments a nice decrease of >50% expression is shown. How do the authors explain this discrepancy?

6) Basal protein levels of VCAM1 are very low in the experiment shown in Figure 3C. There doesn't seem to be a difference in VCAM1 levels between DF and UF and the decrease seen with iCRT5 treatment seems very modest. Is this a representative blot? Stimulating cells with TNFalpha would boost VCAM1 expression and could help see the effect of iCRT5 inhibitor more clearly.

7) For monocyte adhesion assay shown in Figure 3D, please include images that will show HAEC numbers (for example by using DAPI staining), to make sure that the observed decrease in THP1 adhesion is not due to a difference in cell numbers per field of view.

8) Why are some flow experiments conducted for 48 hours and others for 72 hours?
Was there a particular reason not to have the same duration of flow exposure for all experiments for the sake of consistency?

9) Although it is stated that targeting WNT5A leads to 50% reduction in WNT5A mRNA level, on the graph presented in Figure 4C, it seems more like 35-40%.

© 2023. Published by The Company of Biologists under the terms of the Creative Commons Attribution License (https://creativecommons.org/licenses/by/4.0/).

Also, in 2 out of 5 tested samples the knockdown efficiency seems very low (~10%). Is this donor-dependent? Were these samples included in the subsequent functional studies?

10) The authors observed no difference in p-LRP6 in DF compared to UF (Figure 5E). Have they also looked at total levels of LRP6?

11) Have the authors validated the efficiency of the canonical Wnt inhibitor DKK-1 at the used concentration and duration of exposure?

12) Was the efficiency of siRNA targeting RYK validated at mRNA or protein level?

13) Based on images presented in Supplementary Figure S5, the authors conclude that, upon iCRT5 treatment, there is a reduction in transport at bicellular junctions, rather than multicellular junctions. Is it possible to quantify this observation? For example, to quantify proportion of transport at multicellular junctions compared to bicellular junctions in each group?

14) Based on cell staining, the authors conclude ZO-1 expression is increased upon beta-catenin inhibition with iCRT5. Please quantify ZO-1 expression (Figure 7A).

15) The authors have done a lot of elegant mechanistic work to dissect the role of FZD4 and Wnt signaling in regulating endothelial dysfunction in response to atherogenic flow. It would be useful for readers to include a schematic diagram with the proposed pathway, that would include all the inhibitors used and different steps in the pathway that they affect.

Minor:
1) Typo (line 54) should read hemodynamic.
2) Abbreviation UF not introduced in full anywhere.
3) Calnexin size on Western blots is sometimes marked as 90 kDa, sometimes as 100 kDa.
4) Supplementary Figure 2A – remove 3 asterisks on the FZD4 siRNA bar itself.
5) Figure 3C – graph label says iCTR5 instead of iCRT5.
6) Supplementary Figure 4B – representative images for beta-catenin Western blot not shown, although this is stated in the figure legend.
7) Supplementary Figure S4A – main text says that this shows expression of inflammatory markers “when iCRT5 was added for the duration of flow” (which is 72 h), whereas figure legends says iCRT5 was added for the final 24 h of flow exposure – which one is it?
8) Figure 4B WNT5A Western blot image for DF and UF is of poor quality compared to one shown in Figure 4C.
9) Adhere to guidelines regarding human and mouse gene and protein symbols –i.e. capitals italicised for human gene, capitals not italicized for human protein (https://www.genenames.org/, http://www.informatics.jax.org/mgihome/nomen/index.shtml).
10) Figure 4F – main text says cells were exposed to SFRP-1 for 72 h, whereas the corresponding figure legend states SFRP-1 was added for the final 24 h of flow exposure – which one is it?
11) Figure 6A – legend states nuclear stain DRAQ5 is shown, however no nuclear stain can be observed in the images.
12) Please include in Materials and Methods section how you determined the cell shape index (Supplementary Figure 6).
13) Correct Figure 8 labeling (panels and legend do not fit).
14) Lines 256 and 269 should be referring to Figure 8, not 7.
15) Line 257: add “treatment” after “SFRP-1 and IWR-1”.
16) Properly format Descamps et al reference, currently reads “Descamps et al no date” (lines 294 and 296).
17) Include information on used siRNAs in Materials and Methods section (manufacturer, catalogue number).
18) In addition to information provided in the Table of resources, include in the Materials and Methods section the details of treatment with inhibitors (iCRT5, SFRP-1, IWR-1, DKK-1) and what controls were used for these compounds (vehicle – what percentage of DMSO, etc).

Reviewer 2

Advance summary and potential significance to field

© 2023. Published by The Company of Biologists under the terms of the Creative Commons Attribution License (https://creativecommons.org/licenses/by/4.0/).

This manuscript by Rickman et al describes how disturbed flow induces endothelial cell dysfunction via a frizzled-4/beta catenin dependent pathway. This authors use of human aortic endothelial cells (as opposed to HUVECs) make this an interesting and novel study, given the importance of endothelial biology in aortic disease.
The authors also delineate the importance of disturbed flow, rather than low flow which is an important advance for the field.

Comments for the author

This manuscript investigates the importance of disturbed flow in endothelial cell dysfunction and identifies a novel frizzled-4/beta catenin pathway regulating these responses. It is both novel and timely, given the importance of endothelial cells in vascular (aortic) biology/physiology. I do have some comments that will hopefully improve the clarity of the manuscript:
Fig 1B - can a region be magnified to help the reader easily see the difference?

All graphs - please add lines to show what is being statistically compared.

Especially on the graphs with multiple conditions.

SiRNA labelling - the SCR is still an siRNA. Would be clearer to label with the specific target. eg. siFzd4 in Fig 1D, rather than just siRNA.

Can the siRNA qPCRs be repeated with a second independent siRNA? This would control for off target siRNA effects.

Fig 2 - the authors propose a proteasomal pathway. Do levels change with a proteasomal inhibitor? Or is this already accepted in the literature? If so please add a reference.

Fig 3 -
C - The graph doesn't match the WB. There are 2 columns on the graph, but 4 lanes on the WB. Please add a better explanation or show a graph consistent with the WB.
F - can you please show an underexposed b-cat WB for the siFzd4?

In the current format, the presentation of the data is confusing. eg. jumping between PCR and WB. It would be clearer showing the WB images before the analysis graphs. In some figures, the WB panels are different sizes, please make the same size.
In many of the siRNA treatments there is a reduction of cell adhesion proteins (VCAM1) and b-catenin. Can IF be used to show this and confirm cell adhesion structure is not grossly affected. i.e. is this specific? This would make the findings more visual and help the reader.

Finally, a model summarising the pathway would also help the reader.

Minor comment –

I think it would read better by not using the DF/UF abbreviations (especially in the abstract). If you don't agree, please restate the full terms in the results section (lines 101 and 102)

<u>Reviewer 3</u>

Advance summary and potential significance to field

In this manuscript Rickman et al show that disturbed fluid flow across endothelial cells promotes increased expression of frizzled-4 which leads to activation of beta-catenin and expression of pro-inflammatory genes.
Overall this is a nice mechanistic study which provides additional insight into flow-mediated mechanosignaling in endothelial cells.

© 2023. Published by The Company of Biologists under the terms of the Creative Commons Attribution License (https://creativecommons.org/licenses/by/4.0/).

Comments for the author

Major concerns:
1. The rationale for picking frizzled-4 was not clear. Were other frizzled proteins screened? If so can this data be included? If not, perhaps the authors can state why they focused on frizzled-4.
2. I found the morphology differences in Figure 7, with icrt5 treatment striking. Does this suggest that icrt5 treatment causes cells to correctly align in DF as if they were experiencing UF? I am not aware of any treatments that rescues cells in DF to align similar to UF, thus I think this is a very interesting finding that merits some discussion.
3. I found some of the blots to be very weak and of poor contrast.
Specifically 4B, 3C, 2D, 5E, 5H, 5I. I am somewhat concerned about accurate blot analysis when the bands are so weak/faint, as well as the specificity of the antibodies used.
4. The authors did not use ICRT5 treatment with UF, except for in Figure 6 (permeability experiments). This control would help to show if the drug's effects on gene expression are related to frizzled4 signaling or other off target signaling. Likewise some experiments with frizzled4 KD could be performed with UF to see how this affects some of the frizzled4 downstream signaling, to see if it is only occurring during UF as claimed by the authors.

Minor comments:
1. The Gelfand reference is duplicated. Also Descamps reference has no year for the publication.

First revision

Author response to reviewers' comments

We thank all reviewers for taking the time to review our manuscript. We are grateful to each reviewer for providing constructive comments and assisting us in improving our paper. A detailed point-by-point response is provided below. New text is shown as italicised and underlined and new figures included for reference.

Reviewer 1 Advance Summary and Potential Significance to Field...

This manuscript by Rickman and colleagues investigates the role of Frizzled- 4/beta-catenin pathway in regulating endothelial cell dysfunction in response to disturbed flow. The authors find that FZD4 expression is induced by disturbed flow in human aortic cells and mouse aortas and identify an atypical FZD4/beta-catenin pathway which regulates endothelial inflammatory activation and permeability under disturbed flow conditions. The study presents novel findings and provides mechanistic insights into responses of endothelial cells to flow, which are known to be complex and also highly relevant for the development of cardiovascular disease. Overall, this manuscript is interesting, well-written and insightful. The experiments are well- designed and executed and the majority of conclusions are justified by the data presented. However, to be suitable for publication, the authors should address the comments listed below.

Reviewer 1 Comments for the Author...

Major:
1) To better understand the mechanism of FZD4 regulation by flow, the authors should determine whether disturbed flow induces FZD4 expression, or whether undisturbed flow suppresses its expression by testing FZD4 expression in static conditions.

Thank you for suggesting this, we have now performed this analysis and have included a new figure (Figure 1B in revised manuscript). These data demonstrate that DF induces Frizzled-4 expression compared to both static and UF conditions. The text has been updated to highlight this (Pg4, line 106-108):

© 2023. Published by The Company of Biologists under the terms of the Creative Commons Attribution License (https://creativecommons.org/licenses/by/4.0/).

Western blot analysis revealed that disturbed flow (DF) significantly increased the expression of Frizzled-4 relative to undisturbed flow (UF) after 24h and that this increase was sustained for 72h of flow exposure (Figure 1a). Subsequent analysis revealed that DF increases the expression of FZD4 relative to static conditions and UF and that expression levels in EC exposed to static or UF were not significantly different (Figure 1b).

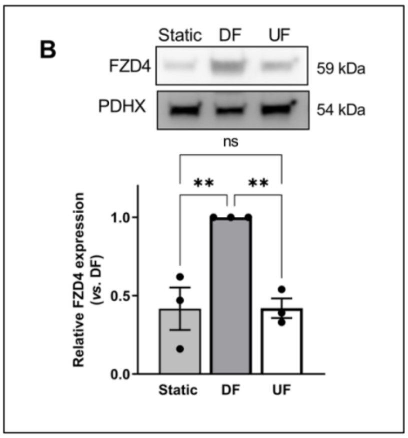

2) The authors should quantify FZD4 expression in the mouse aorta (Figure 1B, en face staining). Were any negative controls used for this staining (for example isotype-matched controls or omission of primary antibody)? I have some doubts about the specificity of this staining, since a strong nuclear signal is observed (indicated by purple colour in the merged images of FZD4 (red) and DAPI (blue)), suggesting nuclear localisation of FZD4 – is this expected?

Thank you for making this suggestion. We have repeated the staining to provide better quality images using a recombinant Frizzled-4 antibody and have conducted the relevant controls (see Figure 1C). We have also quantified the staining as requested (see Figure 1C). We chose to undertake this work in pig tissue as this was readily available to us; moreover, the recombinant antibody cross-reacts only with Frizzled-4 in human, pig and monkey. We have previously conducted CFD analysis in porcine aortas and have shown that this is a robust model for immunostaining of different haemodynamic environments (Serbanovic-Canic 2017). We have updated the methods to outline the tissue collection and staining protocols that were used (Pg 21, line 601-619):

Aortic arches were obtained from juvenile pigs (<16 weeks of age) culled at Newman's abattoir (Farnborough, Surrey) or from The Pirbright Institute (Pirbright, UK). Tissue obtained from The Pirbright Institute was collected in accordance with Directive 2010/63/EU of the European Parliament on the protection of animals used for scientific purposes, under authorisation of the UK Home Office (Project License No. 70/8852) and the Animal Welfare and Ethical Review Board (AWERB) of The Pirbright Institute (Matos, 2022). Pigs were euthanized by overdose of 10ml pentobarbital (Dolethal 200mg/ml solution for injection, Vetoquinol UK Ltd)(Matos, 2022). The aortic arch was excised and fixed immediately in 4% paraformaldehyde for up to 7 days. Aortas were dissected into 1cm^2 sections from either the inner or outer curvature, exposed to disturbed or undisturbed flow according to previous computational fluid dynamic analysis (Serbanovic-Canic, 2017) and the sections were placed into a glass-bottomed 24-well plate. Tissue was permeabilised with Triton X-100 (0.1% v/v) for 5 min before blocking with normal goat serum (10% v/v) for 1 h. It was then stained with β-catenin and FZD4 antibodies (see Table S1) overnight at 4°C for 24 h and 48 h, respectively. Immunostaining was visualised using relevant Alexa Flour 488- or 568-conjugated secondary antibodies. Nuclei were stained using DAPI (1 μg.ml^{-1}) and tissue was imaged en face using a Lecia SP8 confocal microscope with excitation at 405, 488 and 561 nm. A minimum of 4 fields of view were studied for each flow condition using identical laser power and detector gain settings.

© 2023. Published by The Company of Biologists under the terms of the Creative Commons Attribution License (https://creativecommons.org/licenses/by/4.0/).

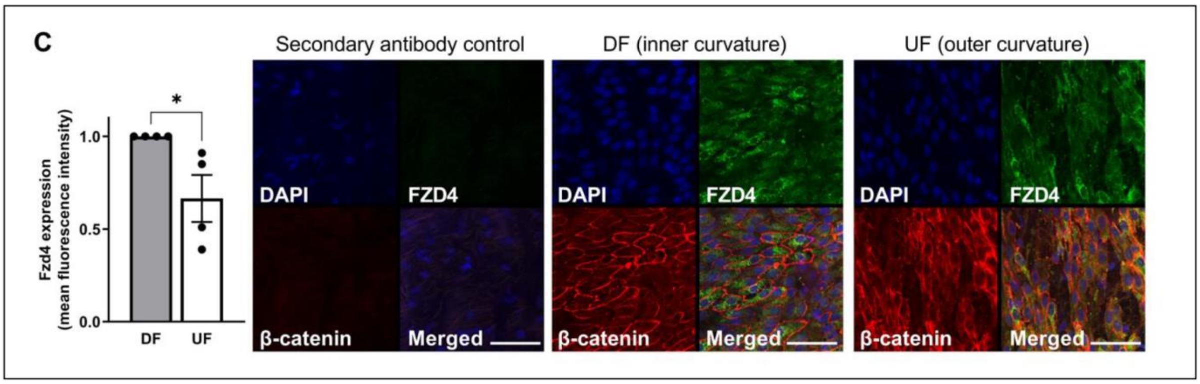

3) The authors apply unpaired or paired t-tests to compare 2 groups where one group (the control) is normalised to 1. T-tests are parametric tests and the assumptions are that the data from each group is normally distributed and that the variance within each group is similar, therefore using a t-test when the values in one group are 1,1,1,1… etc seems inappropriate. A non-parametric test would need to be used in this case or non-normalised data should be used to conduct a t-test.

Thank you for pointing out this oversight. We have re-analysed data where relevant and have included the following text in the methods section (Pg 22, line 629-631): 'Where normalised data are presented, statistical analysis was carried out using a Mann-Whitney test when comparing two conditions or Kruskal-Wallis analysis with Dunn's multiple comparison test for more than two conditions'.

4) What are the area-averaged values of wall shear stress magnitude for the DF and UF regions in this model?
We have added the time-averaged wall shear stress (TAWSS) values for the DF and UF regions to the methods section. DF regions have TAWSS of <0.3 Pa whereas the UF regions has TAWSS of >0.5 Pa.

5) When the authors knock down FZD4 using siRNA (Supplementary Figure 2B), in half of the samples they observe a decrease in FZD4 mRNA expression that is <20% and in one case even an increase in FZD4 mRNA expression in the cells transfected with FZD4 siRNA compared to controls. This seems like an insufficient level of knockdown and doesn't fit FZD4 protein data, where in 5 out of 6 experiments a nice decrease of >50% expression is shown. How do the authors explain this discrepancy?

Fzd4 knockdown using siRNA is transient and we attribute this observation to a lag in the effects on protein vs. message. At 48h post-transfection, expression levels are beginning to increase back to normal levels whilst protein expression remains significantly reduced, as demonstrated in Fig S1A. We have also now removed some of the data points which were erroneously included in the original submission; we now show FZD4 transcript levels in only the experiments where protein knockdown was demonstrated and that were used in the analysis of flow-dependent signaling.

6) Basal protein levels of VCAM1 are very low in the experiment shown in Figure 3C. There doesn't seem to be a difference in VCAM1 levels between DF and UF and the decrease seen with iCRT5 treatment seems very modest. Is this a representative blot? Stimulating cells with TNFalpha would boost VCAM1 expression and could help see the effect of iCRT5 inhibitor more clearly.

Thank you for suggesting this. We have replaced the VCAM-1 western blot data with qPCR data showing the effects of iCRT5 on E-SEL and VCAM-1 expression in the presence of TNFa and added the following text (Pg 7, line 173-174): Treatment with iCRT5 for the final 24h of flow exposure also significantly attenuated the up-regulation of pro-inflammatory genes induced by TNFa (Figure 3c).

© 2023. Published by The Company of Biologists under the terms of the Creative Commons Attribution License (https://creativecommons.org/licenses/by/4.0/).

We have also amended the figure to show the effects of iCRT5 on gene expression in EC exposed to undisturbed flow, as requested by another reviewer. We feel that the THP-1 monocyte adhesion data (now with phase contrast images to show the presence of an intact monolayer; see next comment) nicely illustrate the functional difference in inflammatory activation following inhibition of β-catenin signalling and so have opted to remove the VCAM-1 western blot data from the figure.

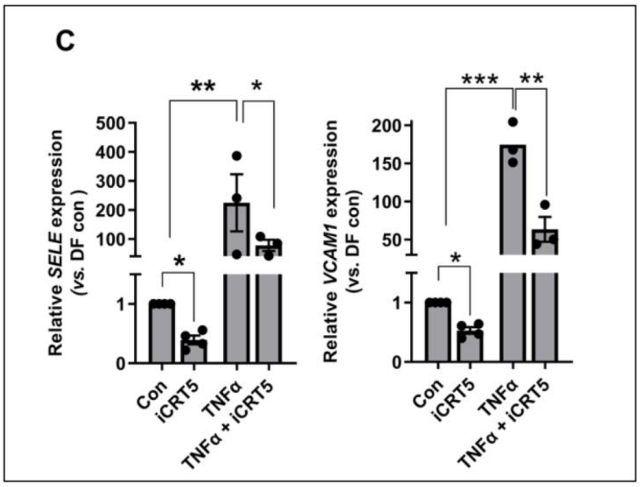

7) For monocyte adhesion assay shown in Figure 3D, please include images that will show HAEC numbers (for example by using DAPI staining), to make sure that the observed decrease in THP1 adhesion is not due to a difference in cell numbers per field of view.
Thank you for highlighting this. We have included phase-contrast images for each condition showing the presence of an intact EC monolayer. Due to space constraints we have removed the iCRT5 72h images.

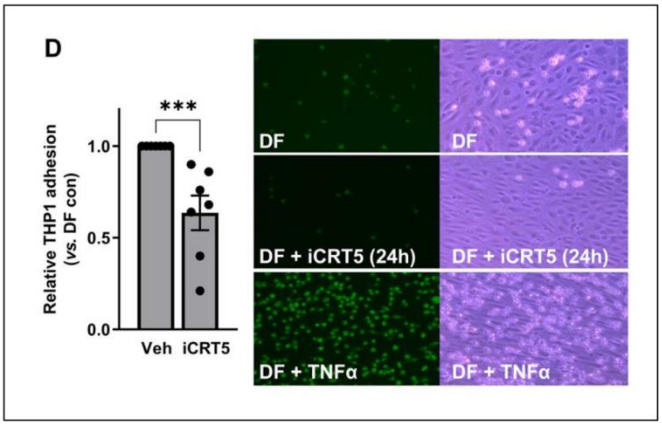

8) Why are some flow experiments conducted for 48 hours and others for 72 hours? Was there a particular reason not to have the same duration of flow exposure for all experiments for the sake of consistency?

Thank you for highlighting the lack of explanation. 48h flow duration was used in experiments where cells were transfected with siRNA to ensure efficacy of knockdown during the flow period. We have added a line to the methods section outlining this difference (Pg 18, line 507-508): For transfection experiments, EC were exposed to flow for 48h to ensure knockdown efficiency.

9) Although it is stated that targeting WNT5A leads to 50% reduction in WNT5A mRNA level, on the graph presented in Figure 4C, it seems more like 35-40%. Also, in 2 out of 5 tested samples the knockdown efficiency seems very low (~10%). Is this donor-dependent? Were these samples included in the subsequent functional studies?

© 2023. Published by The Company of Biologists under the terms of the Creative Commons Attribution License (https://creativecommons.org/licenses/by/4.0/).

Apologies for this error, we have amended the text to state that knockdown efficiency was 35-40%. Inhibition of gene expression did correlate with degree of WNT5A knockdown, but knockdown efficiency was not donor dependent. Samples shown in Fig 4C were included in the functional studies.

10) The authors observed no difference in p-LRP6 in DF compared to UF (Figure 5E). Have they also looked at total levels of LRP6?

Yes, we have now included analysis (and representative blots) demonstrating that total levels of Lrp6 are similar in EC exposed to DF or UF (see Fig 5F in revised version) and have amended the text (Pg 8, line 224-225): Surprisingly, we found no evidence of greater LRP6 phosphorylation under DF than under UF (Figure 5e). Total levels of LRP6 protein were also unchanged (Figure 5f).

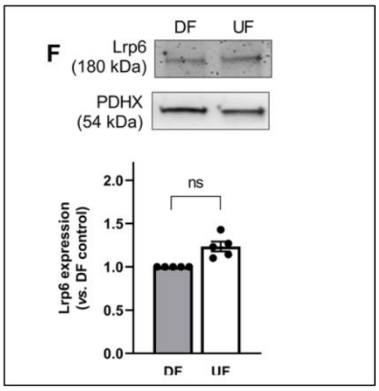

11) Have the authors validated the efficiency of the canonical Wnt inhibitor DKK-1 at the used concentration and duration of exposure?

Our dose regime is appropriate for these cells as we observed changes in the expression of KLF2 and KLF4 at this dose (250ng/ml for 24h); data not shown in the manuscript. This dose has previously been shown to inhibit canonical Wnt signalling e.g. PMID 22105568. We also tested a range of exposure times (72h incubation plus treating with the same dose on consecutive days from 0-72h). DKK-1 was also added in serum-free media to assess whether this had any effect on responses to DF. We saw no effect on EC exposed to DF with any regime despite observing alterations in EC exposed to UF.

12) Was the efficiency of siRNA targeting RYK validated at mRNA or protein level?

Thank you for pointing out this oversight in the original submission. A new figure has been included confirming the knockdown of Ryk at the protein level (Figure 5J) and text added (Pg 9, line 233-234): Transfection with Ryk siRNA resulted in a significant reduction in Ryk expression (Figure 5i)

© 2023. Published by The Company of Biologists under the terms of the Creative Commons Attribution License (https://creativecommons.org/licenses/by/4.0/).

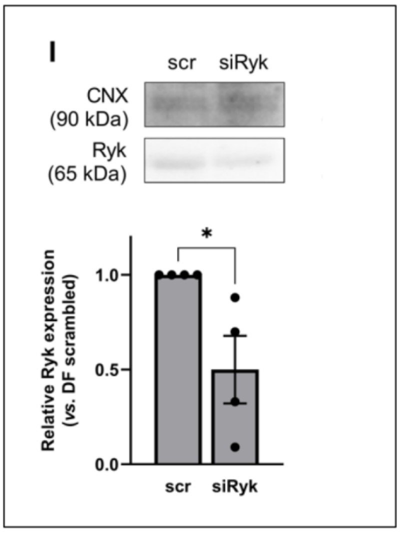

13) Based on images presented in Supplementary Figure S5, the authors conclude that, upon iCRT5 treatment, there is a reduction in transport at bicellular junctions, rather than multicellular junctions. Is it possible to quantify this observation? For example, to quantify proportion of transport at multicellular junctions compared to bicellular junctions in each group?

Whilst it is possible to quantify permeability at tricellular junctions using machine learning methods we do not currently have the resources to perform this analysis. We have altered the text to read 'transport at bicellular junctions appeared to be particularly reduced'

14) Based on cell staining, the authors conclude ZO-1 expression is increased upon beta-catenin inhibition with iCRT5. Please quantify ZO-1 expression (Figure 7A).

We have now included a graph showing quantification of ZO-1 in the presence and absence of iCRT5 supporting these conclusions (Supplementary Figure 5C). A line referring to the measurement of mean fluorescence intensity has also been added to the immunostaining methods section (Pg 20, line 580-581): ZO-1 expression within cell junctions was quantified by measuring mean fluorescence intensity within standardised regions of interest spanning cell junctions

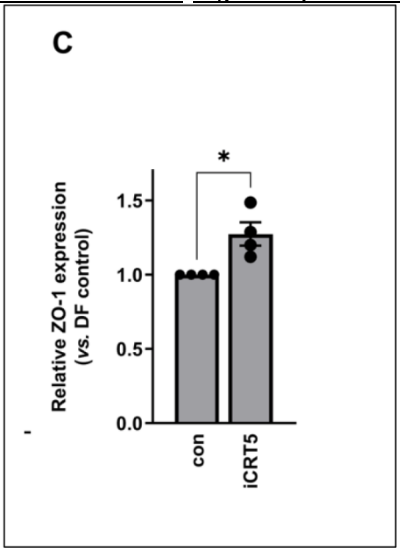

15) The authors have done a lot of elegant mechanistic work to dissect the role of FZD4 and Wnt signaling in regulating endothelial dysfunction in response to atherogenic flow. It would be useful for readers to include a schematic diagram with the proposed pathway, that would include all the inhibitors used and different steps in the pathway that they affect.

© 2023. Published by The Company of Biologists under the terms of the Creative Commons Attribution License (https://creativecommons.org/licenses/by/4.0/).

Thank you for the suggestion, we agree that this is very useful. We had initially prepared a graphical summary but removed it upon submission to meet the figure requirements. We have now reorganised the presentation of the figures to include this schematic diagram (Figure 8).

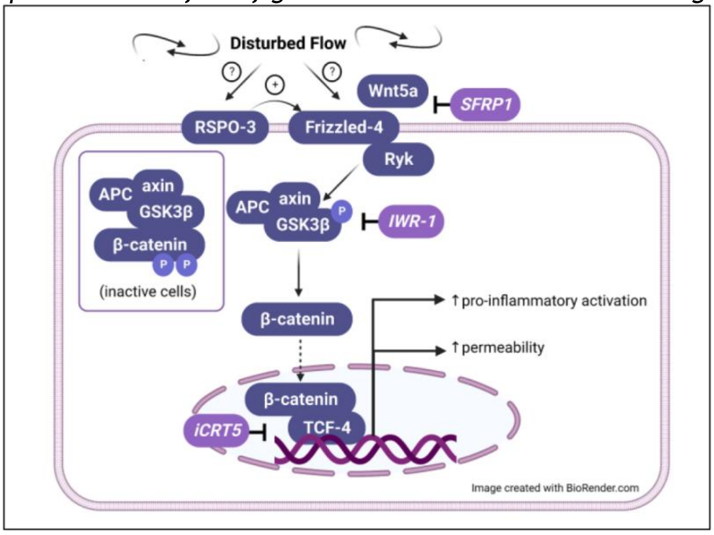

Minor:
1) Typo (line 54) should read hemodynamic.
Corrected!

2) Abbreviation UF not introduced in full anywhere.
This has been corrected and explained before the first use

3) Calnexin size on Western blots is sometimes marked as 90 kDa, sometimes as 100 kDa.
Thank you for spotting this, all bands are now labelled as 90 kDa

4) Supplementary Figure 2A – remove 3 asterisks on the FZD4 siRNA bar itself.
Thank you for spotting this, the asterisks have been removed

5) Figure 3C – graph label says iCTR5 instead of iCRT5.
This graph has now been removed and replaced with data showing the effects of iCRT5 on inflammatory gene expression in EC exposed to undisturbed flow and in the presence of TNFa (see comment above)
6) Supplementary Figure 4B – representative images for beta-catenin Western blot not shown, although this is stated in the figure legend.
Representative western blots have now been included.

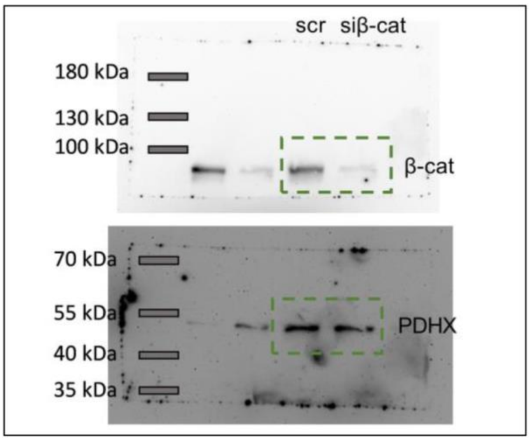

7) Supplementary Figure S4A – main text says that this shows expression of inflammatory markers “when iCRT5 was added for the duration of flow” (which is 72 h), whereas figure legends says iCRT5 was added for the final 24 h of flow exposure – which one is it?

© 2023. Published by The Company of Biologists under the terms of the Creative Commons Attribution License (https://creativecommons.org/licenses/by/4.0/).

The figure legend has been updated to reflect the 72h treatment time

8) Figure 4B WNT5A Western blot image for DF and UF is of poor quality compared to one shown in Figure 4C.
We have added a better resolution image of the WNT5A blot for Fig 4B.

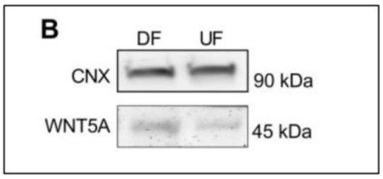

9) Adhere to guidelines regarding human and mouse gene and protein symbols – i.e. capitals italicised for human gene, capitals not italicized for human protein (https://www.genenames.org/, http://www.informatics.jax.org/mgihome/nomen/index.shtml).
We have amended the formatting of gene and protein names throughout the manuscript.

10) Figure 4F – main text says cells were exposed to SFRP-1 for 72 h, whereas the corresponding figure legend states SFRP-1 was added for the final 24 h of flow exposure.
This figure legend has been updated to reflect the 72h treatment time.

11) Figure 6A – legend states nuclear stain DRAQ5 is shown, however no nuclear stain can be observed in the images.
The reference to DRAQ5 has been removed.

12) Please include in Materials and Methods section how you determined the cell shape index (Supplementary Figure 6).
A line has been added on methodology and the y-axis updated to more accurately reflect what was measured (length-to-width ratio). Please note that this figure now appears in Supplementary Figure 5.

13) Correct Figure 8 labeling (panels and legend do not fit).
All labelling should now be correct. Please note this is now Figure 7 in the revised version.

14) Lines 256 and 269 should be referring to Figure 8, not 7.
Figures 6, 7 and 8 from the original submission have been altered to include a graphical summary (now Figure 8). The correct labelling is now shown for this data.

15) Line 257: add "treatment" after "SFRP-1 and IWR-1".
This has been added.

16) Properly format Descamps et al reference, currently reads "Descamps et al, no date" (lines 294 and 296).
This has now been formatted correctly and the details in the reference list corrected.

17) Include information on used siRNAs in Materials and Methods section (manufacturer, catalogue number).
This information has been added to the supplementary table of resources and additional details included in the methods text.

18) In addition to information provided in the Table of resources, include in the Materials and Methods section the details of treatment with inhibitors (iCRT5, SFRP-1, IWR-1, DKK-1) and what controls were used for these compounds (vehicle – what percentage of DMSO, etc).
A new paragraph including these details has been added to the Methods section. The supplementary file has also been updated to include details of the controls (Pg 17, 476- 480).

© 2023. Published by The Company of Biologists under the terms of the Creative Commons Attribution License (https://creativecommons.org/licenses/by/4.0/).

In some experiments, cell were treated with the following inhibitors: iCRT5 (50 µM; Abcam), huDKK-1 (250 ng.ml^{-1}; R&D Systems) or huSFRP-1 (100 ng.ml^{-1}; Peprotech). Inhibitors were added for the duration of flow exposure (72h) or for the last 24h of flow exposure (24h) as indicated below. DMSO (0.1% v/v), PBS (0.25% v/v) or H_2O (0.2% v/v) was used as a vehicle control, respectively. Additional details are included in Table S1.

Reviewer 2 Advance Summary and Potential Significance to Field...

This manuscript by Rickman et al describes how disturbed flow induces endothelial cell dysfunction via a frizzled-4/beta catenin dependent pathway. This authors use of human aortic endothelial cells (as opposed to HUVECs) make this an interesting and novel study, given the importance of endothelial biology in aortic disease.
The authors also delineate the importance of disturbed flow, rather than low flow, which is an important advance for the field.

Reviewer 2 Comments for the Author...

This manuscript investigates the importance of disturbed flow in endothelial cell dysfunction and identifies a novel frizzled-4/beta catenin pathway regulating these responses. It is both novel and timely, given the importance of endothelial cells in vascular (aortic) biology/physiology. I do have some comments that will hopefully improve the clarity of the manuscript:
Fig 1B - can a region be magnified to help the reader easily see the difference?

We have repeated these experiments in porcine aorta and now show images at a higher magnification.

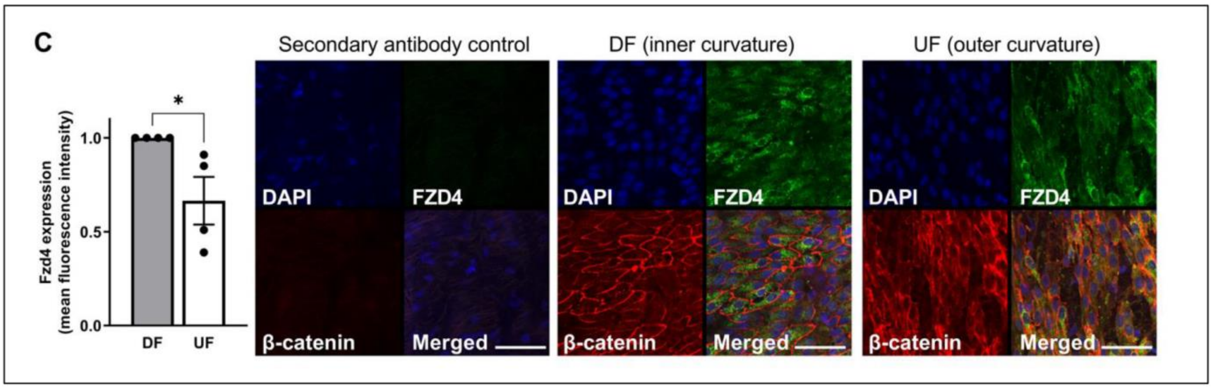

All graphs - please add lines to show what is being statistically compared. Especially on the graphs with multiple conditions.

Thank you for this suggestion. We have added lines to make the statistical analysis clearer on all graphs.

SiRNA labelling - the SCR is still an siRNA. Would be clearer to label with the specific target. eg. siFzd4 in Fig 1D, rather than just siRNA.

Thank you for this suggestion, all graphs have been re-labelled as siFzd4, siRSPO-3 etc.

Can the siRNA qPCRs be repeated with a second independent siRNA? This would control for off target siRNA effects.

Unfortunately we do not have the resources to carry out additional siRNA knockdowns, however we believe that a significant strength of this study is the use of siRNA targeting different components of the FZD4-B-catenin pathway in combination with a number of small molecule inhibitors that show similar effects.

© 2023. Published by The Company of Biologists under the terms of the Creative Commons Attribution License (https://creativecommons.org/licenses/by/4.0/).

Fig 2 - the authors propose a proteasomal pathway. Do levels change with a proteasomal inhibitor? Or is this already accepted in the literature? If so, please add a reference.

Apologies, this should have read 'FZD4 protein expression can be regulated by lysosomal degradation'- this has now been amended. References are also included to support this statement:
*Koo (2012) 'Tumour suppressor RNF43 is a stem-cell E3 ligase that induces endocytosis of Wnt receptors', Nature, **488**(7413), 665-669*
*Hao (2012) 'ZNRF3 promotes Wnt receptor turnover in an R-spondin-sensitive manner', Nature, **485**(7397), 195-200.*
*Hao (2016) 'Control of Wnt Receptor Turnover by R-spondin-ZNRF3/RNF43 Signaling Module and Its Dysregulation in Cancer', Cancers, **8**(6).*
Fig 3 -C - The graph doesn't match the WB. There are 2 columns on the graph, but 4 lanes on the WB. Please add a better explanation or show a graph consistent with the WB.

Thank you for pointing this out. We have now removed the VCAM-1 western blot data from the figure to allow additional data to be presented (see comments from other reviewers). We feel that the THP-1 monocyte adhesion data (now with phase contrast images to show the presence of an intact monolayer) nicely illustrates the functional difference in inflammatory activation following inhibition of β- catenin signalling and so have opted to remove the VCAM-1 western blot data from the figure.

F - can you please show an underexposed b-cat WB for the siFzd4?

The blot has been replaced

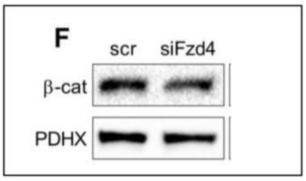

In the current format, the presentation of the data is confusing. eg. jumping between PCR and WB. It would be clearer showing the WB images before the analysis graphs. In some figures, the WB panels are different sizes, please make the same size.

Thank you for pointing this out, where necessary, figures have been rearranged to display the western blot images above the corresponding graphs. All western blot images are also now displayed at a standardised size.

In many of the siRNA treatments there is a reduction of cell adhesion proteins (VCAM1) and b-catenin. Can IF be used to show this and confirm cell adhesion structure is not grossly affected. i.e. is this specific? This would make the findings more visual and help the reader.

We feel that the immunofluorescence images shown throughout the manuscript demonstrate nicely that cell adhesion and confluence is maintained (e.g. Figure 7 shows IF with siFzd4 and siWnt5a compared to scrambled controls). It is clear that there is no difference in overall structure and integrity of the endothelium (except for the junctional structure which is discussed extensively).

Finally, a model summarising the pathway would also help the reader.
Thank you for the suggestion, we agree that this is very useful. We had initially prepared a graphical summary but removed it upon submission to meet the figure requirements. We have now reorganised the presentation of the figures to include this schematic diagram (Figure 8).

© 2023. Published by The Company of Biologists under the terms of the Creative Commons Attribution License (https://creativecommons.org/licenses/by/4.0/).

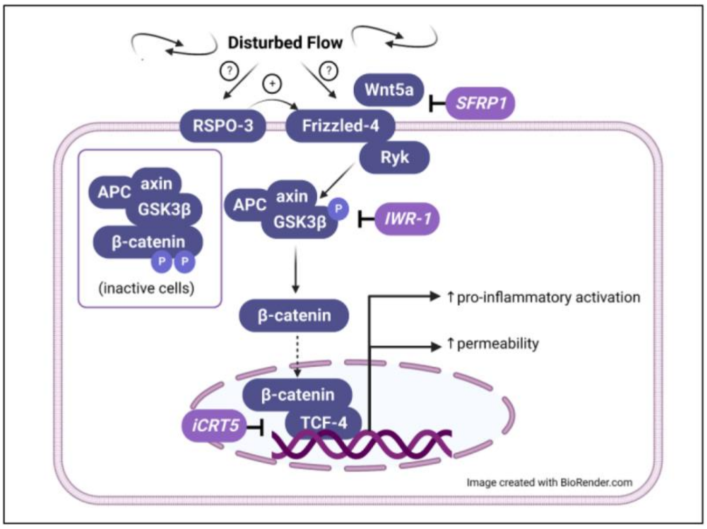

Minor comment -

I think it would read better by not using the DF/UF abbreviations (especially in the abstract). If you don't agree, please restate the full terms in the results section (lines 101 and 102)
I have removed the abbreviations from the abstract as suggested. Full terms have been restated in lines 104-105 in the revised version.

Reviewer 3 Advance Summary and Potential Significance to Field...

In this manuscript Rickman et al show that disturbed fluid flow across endothelial cells promotes increased expression of frizzled-4 which leads to activation of beta-catenin and expression of pro-inflammatory genes. Overall this is a nice mechanistic study which provides additional insight into flow-mediated mechanosignaling in endothelial cells.

Reviewer 3 Comments for the Author...

Major concerns:
1. The rationale for picking frizzled-4 was not clear. Were other frizzled proteins screened? If so can this data be included? If not, perhaps the authors can state why they focused on frizzled-4.
We have included a sentence to highlight that the rationale for studying Frizzled-4 was based on the observation that it was found to be differentially expressed in a transcriptomic analysis of flow-sensitive genes in the porcine aorta.

Frizzled-4 (FZD4) mRNA levels were previously shown to be sensitive to flow conditions in a transcriptomic analysis of the porcine aorta (Serbanovic-Canic, 2017). (Pg 4, line 101-102)

2. I found the morphology differences in Figure 7, with icrt5 treatment striking. Does this suggest that icrt5 treatment causes cells to correctly align in DF as if they were experiencing UF? I am not aware of any treatments that rescues cells in DF to align similar to UF, thus I think this is a very interesting finding that merits some discussion.
Thank you for highlighting this important finding. We observed similar effects in a previous study (Warboys et al ATVB 2014) following application of resveratrol. We have now added the following to the manuscript (Pg 15, line 419-422): 'We observed similar alterations to EC morphology under DF following treatment with resveratrol (Warboys, 2014). Future studies should investigate whether inhibition of β-catenin signalling can switch cells exposed to atherogenic DF to an atheroprotective phenotype'.

© 2023. Published by The Company of Biologists under the terms of the Creative Commons Attribution License (https://creativecommons.org/licenses/by/4.0/).

3. I found some of the blots to be very weak and of poor contrast. Specifically 4B, 3C, 2D, 5E, 5H, 5I. I am somewhat concerned about accurate blot analysis when the bands are so weak/faint, as well as the specificity of the antibodies used.
We have replaced 2D, 4B, 5E, 5H and 5I with better quality images. 3C has been removed from the manuscript and replaced with new data (as requested by other reviewers)

4. The authors did not use ICRT5 treatment with UF, except for in Figure 6 (permeability experiments). This control would help to show if the drug's effects on gene expression are related to frizzled4 signaling or other off target signaling. Likewise some experiments with frizzled4 KD could be performed with UF to see how this affects some of the frizzled4 downstream signaling, to see if it is only occurring during UF as claimed by the authors.

Thank you for raising this. We have now included additional data within the manuscript demonstrating that the effects of iCRT5 and Fzd4 knockdown are specific to EC exposed to disturbed flow. We demonstrate that the expression of proinflammatory genes, E-SEL, MCP-1 and *VCAM-1 are reduced in EC exposed to UF (as expected) and that this is not significantly altered following inhibition of the Fzd4 signalling pathway (see Figure 1d and 3b). We have also emphasised this finding within the manuscript text.*

Pg 5, line 120-122 This effect was specific to EC exposed to DF. As expected, the expression of pro-inflammatory genes was significantly lower in EC exposed to UF and expression levels were not altered following FZD4 knockdown

Pg 6, line 170-171 The addition of iCRT5 had no effect on pro-inflammatory gene expression in EC exposed to UF (Fig. 3b).

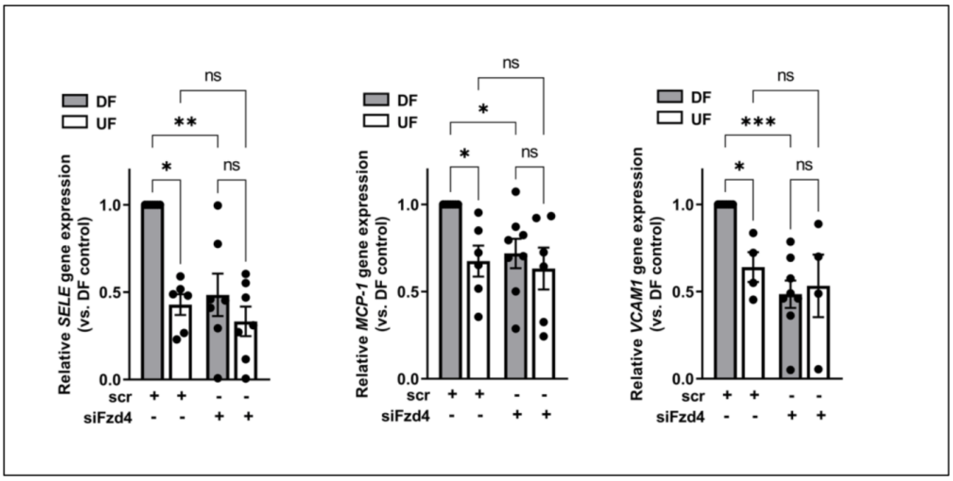

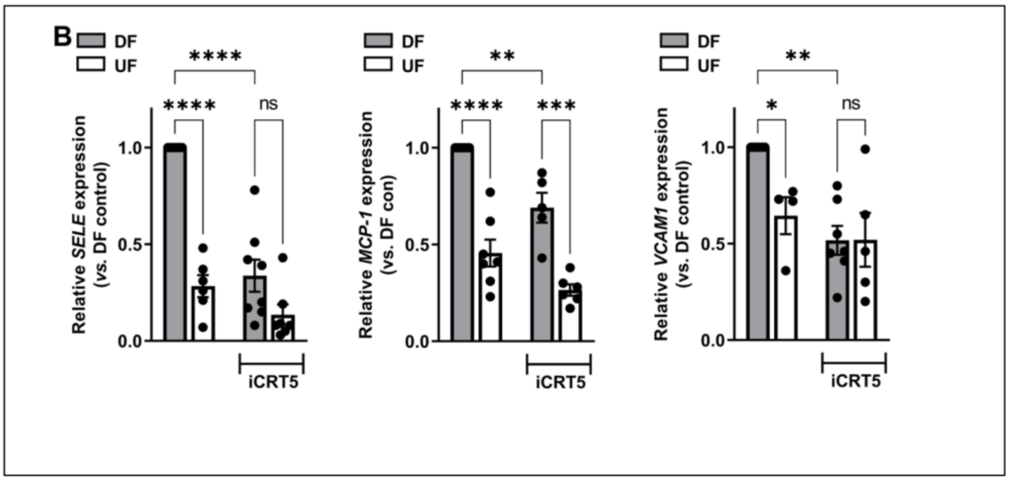

© 2023. Published by The Company of Biologists under the terms of the Creative Commons Attribution License (https://creativecommons.org/licenses/by/4.0/).

Minor comments:
1. The Gelfand reference is duplicated. Also Descamps reference has no year for the publication.
The duplicated reference has been removed. The Descamps reference has now been formatted correctly and the details in the reference list corrected.

Minor changes, not presented here, were incorporated to correct other typographical errors and improve clarity

---

Second decision letter

MS ID#: JOCES/2022/260449

MS TITLE: Disturbed flow increases endothelial inflammation and permeability via a Frizzled-4-ß-catenin-dependent pathway

AUTHORS: Matthew Rickman, Mean Ghim, Kuin Pang, Ana Cristina von Huelsen-Rocha, Elena M Drudi, Macià Sureda-Vives, Nicolas Ayoub, Virginia Tajadura-Ortega, Sarah J George, Peter D Weinberg, and Christina M Warboys
ARTICLE TYPE: Research Article

We have now reached a decision on the above manuscript.

To see the reviewers' reports and a copy of this decision letter, please go to: https://submit-jcs.biologists.org and click on the 'Manuscripts with Decisions' queue in the Author Area. (Corresponding author only has access to reviews.)

As you will see, the reviewers gave favourable reports but Reviewer 1 identified a few specific issues to address in the manuscript. Please review and respond to all of these issues and clearly explain in the cover letter and response to reviews how you have done so. I hope that you will be able to carry these out because I would like to be able to accept your paper.

Please ensure that you clearly highlight all changes made in the revised manuscript. Please avoid using 'Tracked changes' in Word files as these are lost in PDF conversion.

I should be grateful if you would also provide a point-by-point response detailing how you have dealt with the points raised by the reviewers in the 'Response to Reviewers' box. Please attend to all of the reviewers' comments. If you do not agree with any of their criticisms or suggestions please explain clearly why this is so.

Reviewer 1

Advance summary and potential significance to field

The authors have addressed all my comments satisfactorily.

Comments for the author

Minor comments:
1) Statistical tests - the authors have re-analysed the data in most cases however there are still a few instances where t-test has been inappropriately used, please amend:
- Figure 5 (C, D)
- Figure 7 (E)
- Supp. Figure 2 (A, B)

© 2023. Published by The Company of Biologists under the terms of the Creative Commons Attribution License (https://creativecommons.org/licenses/by/4.0/).

2) Make sure n numbers are correct in all figure legends, I have noticed a couple of discrepancies between the graph and the corresponding figure legend - examples Figure 1 (A), Figure 4 (A).
3) Line 206 should be Fig. S6 not Fig. S7.
4) Same line (206) - VCAM1 should be taken out as the difference between control and SFRP-1 treatment wasn't statistically significant, according to the data presented in Figure S6A.
5) Line 313 - correct "proteasomal" to "lysosomal".

Reviewer 2

Advance summary and potential significance to field

The authors have addressed all my concerns and I am happy to recommend this manuscript for publication. I agree with the authors that extra siRNAs are not required as a pharmacological approach is also employed to back up the siRNA results.

Congratulations on what is a novel, important and timely manuscript.

Comments for the author

None - this revised manuscript is now acceptable for publication in my opinion.

Reviewer 3

Advance summary and potential significance to field

In this manuscript Rickman et al show that disturbed fluid flow across endothelial cells promotes increased expression of frizzled-4 which leads to activation of beta-catenin and expression of pro-inflammatory genes. Overall this is a nice mechanistic study which provides additional insight into flow-mediated mechanosignaling in endothelial cells. An especially striking observation is that modulation of beta-catenin signaling can change cells in a disturbed flow environment into a phenotype that mirrors the phenotype in atheroprotective flow.

Comments for the author

I feel the authors have fully addressed my prior concerns.

Second revision

Author response to reviewers' comments

We would like to thank all reviewers for appraising our manuscript following revision and for the positive feedback and constructive comments. We have made amendments to the manuscript as highlighted by reviewer 1 (detailed response included below).

Reviewer 1

Advance Summary and Potential Significance to Field:
The authors have addressed all my comments satisfactorily.

© 2023. Published by The Company of Biologists under the terms of the Creative Commons Attribution License (https://creativecommons.org/licenses/by/4.0/).

Reviewer 1 Comments for the Author:

Minor comments:

1) Statistical tests - the authors have re-analysed the data in most cases, however there are still a few instances where t-test has been inappropriately used, please amend:
- Figure 5 (C, D)
- Figure 7 (E)
- Supp. Figure 2 (A, B)

Thank you for spotting this, I appear to have failed to update these figure legends with the correct statistical tests during revision. All figure legends now contain the correct information (Fig 5C, 5D, 7E, S2A and S2B were analysed by Mann-Whitney test).

2) Make sure n numbers are correct in all figure legends, I have noticed a couple of discrepancies between the graph and the corresponding figure legend - examples Figure 1 (A), Figure 4 (A).

Thank you for pointing this out – I have been through all figure legends (including the supplementary file) and all n numbers are now correct – changes are highlighted in manuscript.

3) Line 206 should be Fig. S6 not Fig. S7.
Thank you for spotting this – the text has been changed to read Fig. S6.

4) Same line (206) - VCAM1 should be taken out as the difference between control and SFRP-1 treatment wasn't statistically significant, according to the data presented in Figure S6A.

Thank you for highlighting this. We have removed VCAM1 from this sentence and added a note about VCAM1 expression following 72h inhibition as it was significantly altered under these conditions. The text now reads;
'SFRP-1 treatment for 24h significantly reduced the expression of SELE and MCP-1 in EC exposed to DF (Fig. S6). Interestingly, inhibition was greater when cells were exposed to SFRP1 for 72h and the expression of VCAM1 was also significantly reduced (Fig. 4F)'

5) Line 313 - correct "proteasomal" to "lysosomal".
Thank you for spotting this, the text has now been corrected.

Reviewer 2

Advance Summary and Potential Significance to Field:
The authors have addressed all my concerns and I am happy to recommend this manuscript for publication. I agree with the authors that extra siRNAs are not required as a pharmacological approach is also employed to back up the siRNA results.

Congratulations on what is a novel, important and timely manuscript.

Reviewer 2 Comments for the Author:
None - this revised manuscript is now acceptable for publication in my opinion.

Reviewer 3

Advance Summary and Potential Significance to Field:
In this manuscript Rickman et al show that disturbed fluid flow across endothelial cells promotes increased expression of frizzled-4 which leads to activation of beta-catenin and expression of pro-inflammatory genes. Overall this is a nice

© 2023. Published by The Company of Biologists under the terms of the Creative Commons Attribution License (https://creativecommons.org/licenses/by/4.0/).

mechanistic study which provides additional insight into flow-mediated mechanosignaling in endothelial cells. An especially striking observation is that modulation of beta-catenin signaling can change cells in a disturbed flow environment into a phenotype that mirrors the phenotype in atheroprotective flow.

Reviewer 3 Comments for the Author:
I feel the authors have fully addressed my prior concerns.

Third decision letter

MS ID#: JOCES/2022/260449

MS TITLE: Disturbed flow increases endothelial inflammation and permeability via a Frizzled-4-ß-catenin-dependent pathway

AUTHORS: Matthew Rickman, Mean Ghim, Kuin Pang, Ana Cristina von Huelsen-Rocha, Elena M Drudi, Macià Sureda-Vives, Nicolas Ayoub, Virginia Tajadura-Ortega, Sarah J George, Peter D Weinberg, and Christina M Warboys
ARTICLE TYPE: Research Article

I am happy to tell you that your manuscript has been accepted for publication in Journal of Cell Science, pending standard ethics checks.

© 2023. Published by The Company of Biologists under the terms of the Creative Commons Attribution License (https://creativecommons.org/licenses/by/4.0/).
